# NOD2 in monocytes negatively regulates macrophage development through TNFalpha

**DOI:** 10.3389/fimmu.2023.1181823

**Published:** 2023-06-21

**Authors:** Camille Chauvin, Daniel Alvarez-Simon, Katarina Radulovic, Olivier Boulard, William Laine, Myriam Delacre, Nadine Waldschmitt, Elodie Segura, Jérome Kluza, Mathias Chamaillard, Lionel F. Poulin

**Affiliations:** ^1^ U1019, Institut Pasteur de Lille, Univ. Lille, Centre National de la Recherche Scientifique, Inserm, Centre Hospitalo- Universitaire Lille, Lille, France; ^2^ INSERM U1138, Centre de Recherche des Cordeliers, Paris, France; ^3^ Unité de Recherche Clinique, Centre Hospitalier de Valenciennes, Valenciennes CEDEX, France; ^4^ U1003, Univ. Lille Inserm, Lille, France; ^5^ UMR9020-U1277 - CANTHER - Cancer Heterogeneity Plasticity and Resistance to Therapies, University Lille, Lille, France; ^6^ Chair of Nutrition and Immunology, School of Life Sciences, Technische Universität München, Freising-Weihenstephan, Germany; ^7^ INSERM U932, Institut Curie, Paris Sciences et Lettres Research University, Paris, France

**Keywords:** NOD2, monocytes, macrophages, mo-DCs, TNF-α, colitis, microbiota, mTOR

## Abstract

**Objective:**

It is believed that intestinal recruitment of monocytes from Crohn’s Disease (CD) patients who carry NOD2 risk alleles may repeatedly give rise to recruitment of pathogenic macrophages. We investigated an alternative possibility that NOD2 may rather inhibit their differentiation from intravasating monocytes.

**Design:**

The monocyte fate decision was examined by using germ-free mice, mixed bone marrow chimeras and a culture system yielding macrophages and monocyte-derived dendritic cells (mo-DCs).

**Results:**

We observed a decrease in the frequency of mo-DCs in the colon of *Nod2*-deficient mice, despite a similar abundance of monocytes. This decrease was independent of the changes in the gut microbiota and dysbiosis caused by Nod2 deficiency. Similarly, the pool of mo-DCs was poorly reconstituted in a *Nod2*-deficient mixed bone marrow (BM) chimera. The use of pharmacological inhibitors revealed that activation of NOD2 during monocyte-derived cell development, dominantly inhibits mTOR-mediated macrophage differentiation in a TNFα-dependent manner. These observations were supported by the identification of a TNFα-dependent response to muramyl dipeptide (MDP) that is specifically lost when CD14-expressing blood cells bear a frameshift mutation in NOD2.

**Conclusion:**

NOD2 negatively regulates a macrophage developmental program through a feed-forward loop that could be exploited for overcoming resistance to anti-TNF therapy in CD.

## Introduction

Monocytes and macrophages play an essential role in the immune system due to their ability to convert from a homeostatic to a pro-inflammatory phenotype in response to immune invasion. Circulating monocytes continuously replenish the intestinal populations of macrophages during homeostasis. The intestine is particularly enriched in phagocytes that ensure robustness in the steady state and play a role in processes of remodeling upon tissue injury. Among those, some macrophages have been seeded embryonically and self-renewed from yolk sac-derived precursor cells in mice. Under healthy conditions, intestinal macrophages have an estimated half-life of three weeks. In human and mouse, subsets of macrophages have been characterized based on a differential expression of CD11c (1, 2). In mice, the pool of Ly6C^low^ CX3CR1^int^ macrophages that share some features of monocyte-derived dendritic cells (mo-DCs) is then continually replenished by the emigration of short-lived Ly6C^high^ monocytes from the bloodstream (3). Activation of the C-C chemokine receptor type 2 (CCR2) is a prerequisite for such leukocytes to exit from the bone marrow at steady state (4). Along a continuum of differentiation stages, Ly6C^high^ monocytes may then give rise to nascent phagocytes with ascribed different functions and distinct bioenergetic programs (5). Whereas mature macrophages are largely immotile with phagocytic capacity (6), mo-DCs are believed to migrate for presenting protein antigens on major histocompatibility complexes class I and II (MHCI and MHCII) molecules to T cells (7–9). The community of circulating Ly6C^high^ monocytes that are rapidly mobilized upon injury is then thought to seemingly serve as a reservoir of mo-DCs that can be distinguished from CD11c-expressing macrophages on the basis of ontogenetic, morphological, and gene expression criteria (10).

In humans, the equivalent of inflammatory monocytes is classical monocytes CD14^+^CD16^-^ which represent up to 80-95% of the large reservoir of monocytes. They can be distinguished from intermediate and non-classical subsets by their expression of well-characterized surface proteins, including CD16 (also referred as Fc gamma receptor IIIa) and the glycoprotein CD14 that acts as a co-receptor for toll-like receptor 4. While the intermediate monocytes CD14^+^CD16^+^ regulate angiogenesis and modulate effector T cell activity (11), the nonclassical monocytes CD14^-^CD16^+^ are mobile and involved in the maintenance of vascular homeostasis (12). Recent studies demonstrated that the molecular ontogeny of human monocyte-derived cells is orchestrated by distinct transcription factors that are specifically activated by environmental cues. Comparative transcriptomic analysis revealed that the monocyte fate specification into mo-DC and monocyte-derived macrophages (mo-Mac) is at least partially coordinated by Interferon Regulatory Factor 4 (IRF4) and MAF BZIP Transcription Factor B (MAFB) (8) respectively.

As monocyte-derived cells display high plasticity to their environment, they can express high susceptibility to the chronic inflammatory stimulations arising in the intestine during inflammatory bowel diseases (IBD). The inflammatory microenvironment, including increasing levels of CCL2 and IL-8, promotes the recruitment of monocytes in the intestine of IBD patients (13, 14). While an increase of immature macrophages has been correlated with the endoscopic severity score of the disease (15), a defect of mo-DCs function, including a decrease in TH17 activation and IL-12 production in response to NOD2 stimulation has been observed in Crohn’s disease (CD) patients (16, 17). In IBD, macrophages have been associated with inflammation and fibrosis while (cDCs) express a more pro-inflammatory phenotype (18). Of particular importance, it has now been elegantly demonstrated that the monocyte fate toward mo-DCs is orchestrated by the aryl hydrocarbon receptor (8), which defect is associated with a susceptibility to several common diseases, including CD (19). Likewise, monocytes may give rise to mo-DCs upon inhibition of the mammalian target of rapamycin (mTOR) pathway (20) for which the sustained activation in CD is likely a consequence of a genetically predisposed defect in autophagy (21). Interestingly, an increased number of inflammatory macrophages is observed within the intestinal mucosa of CD patients at the expense of their pro-resolving counterpart (22). Those data were supported by a recent single-cell analysis of inflamed tissues from CD, which revealed the presence of a discrete subset of pathogenic macrophages within the diseased intestine of CD patients that fail to respond to anti-TNF therapy (23). It is thereby tempting to speculate that a defect in the development of monocyte-derived phagocytes may allow the expansion of pathogenic macrophages that maintain a TH1-biased CD4 T cell response through the production of inflammatory and fibrogenic effectors.

Genetic variants in the *NOD2* gene confer an increased susceptibility to CD, likely due to the loss of NOD2 function. Furthermore, patients who carry NOD2 risk alleles are at greater risk of developing stricturing disease, that corresponds to a narrowing of the intestine due to continued inflammation (23). The Nucleotide-binding Oligomerization Domain (NOD)-like receptor NOD2 is a cytosolic sensor of bacterial muramyl dipeptide (MDP). MDP is an active component in Freund’s complete adjuvant and derivatives have been synthesized for improving their pharmacological properties. The recognition of MDP by NOD2 has been associated with autophagy induction, bacterial destruction, and antigen presentation in DC. Indeed, while NOD2-mediated autophagosome formation was necessary for MHC II upregulation, mo-DCs from CD–variant NOD2 patients were unable to kill and localize intact *Escherichia coli* into the lysosomal compartment; however, this defect was reversible with rapamycin (24). Furthermore, probiotics induce an anti-inflammatory phenotype on bone marrow-derived DC with an increase of IL-10 production in a Nod2- and a strain-specific manner in a mouse model of TNBS-mediated colon inflammation (25). While peptidoglycan derived from the *L. salivarius* Ls33 strain only partially activated DC *in vitro*, it induced protection associated with an increase of IL-10 production and of regulatory CD11c^+^CD103^+^ DCs and CD4^+^FoxP3^+^ Treg cells in the mesenteric lymph nodes of colitic mice.

Despite substantial efforts that were made in studying how the homeostatic trafficking of monocytes is controlled by NOD2, it remains unclear whether NOD2 may orchestrate their differentiation into a developmentally distinct subset of cells that are specialized for the maintenance of immune surveillance. After stimulating their exit from the bone marrow, classical monocytes have the capacity of being converted into non-classical cells in a NOD2-dependent manner (26). It has been proposed that MDP might increase the exit of monocytes from bone marrow and the yield of Ly6C^lo^ in the blood of WT but not *Nod2* ^-/-^ mice (26). In addition, NOD2 has been involved in the CCL2 production by colonic stromal cells in *Citrobacter rodentium* infection (27). Besides this phenomenon, it is now well established that sensing of bacterial endotoxin promotes the mobilization of inflammatory monocytes, which can develop into cells with a typical probing morphology and with critical features of mo-DCs including cross-priming capacities of cell-associated antigens to CD8^+^ T cells (7). It suggests the likelihood that loss of NOD2 may directly inhibit the development of potentially reprogrammable cells of monocytic origin into inflammatory macrophages. NOD2 has been involved in the induction of immune tolerance *via* the generation of immature CD103^+^ classical dendritic cells (cDC1) associated with tolerogenic DC, in a GM-CSF-dependent manner in mice (28). In addition, the cross-tolerization to multiple TLRs has been observed after chronic stimulation of NOD2 with MDP (29), and as a result, increased activation of TLR signaling has been proposed in the process of NOD2 deficiency-induced intestinal epithelium inflammation. In parallel, this concept reminds the one of “trained immunity” upon parasite infection where chromatin remodeling leads to the induction of instructed immune responses by monocytes or macrophages (30). The context-dependence of differentiation has already been observed in monocytic cells. In another setting, programmable cells of monocytic origin (PCMOs) with plastic properties have been described to give rise to two subsets of DC in the presence of IL-3 or TNF-α (31). While mo-DCs are markedly less abundant within the healthy intestine than macrophages, it does not exclude the possibility that mo-DCs may play an essential role in intestinal homeostasis. Indeed, DCs have been shown to play a crucial role in gut homeostasis by interacting with the gut microbiome and by regulating the balance between TH1/TH17 and Tregs (32). Mononuclear phagocytes (MNPs), including monocytes, macrophages, and dendritic cells (DCs), are present in large numbers in the colonic lamina propria and fulfill a variety of overlapping functions that are critical to the maintenance of gut homeostasis. Disruption of the intestinal MNP system leads to infection and inflammation (33–39). In general, mo-DCs can enhance the ability of classical DCs to elicit adaptive responses by presenting antigens to T cells directly in tissues to increase their effector functions (40–42). Distinct tolerogenic DCs have been identified in different anatomic parts of the intestine suggesting region-specific mechanisms of homeostasis (32). It is yet unclear whether mo-DCs and conventional DCs are complementary or redundant in the maintenance of gut homeostasis (43). The recognition of Pattern Recognition Receptors (PRR) and C-type lectin receptors by DCs has been shown to influence their metabolic reprogramming toward glycolysis, and eventually to shape their contribution to immune responses or tolerance (43). MDP has been reported to induce rapid metabolic reprogramming in human macrophages (44). Murabutide, a NOD2 ligand, and TNF-α have been reported to promote the differentiation of mo-DCs while the TLR2 agonist Pam3Csk4 assisted mo-Macs development in a culture of human monocytes with M-CSF, IL-4, and TNF-α, and in mouse skin *in vivo* (9). The involvement of the mTOR pathway has been described in the differentiation of mo-DCs. However, while the differentiation and the survival of human mo-DCs are conditioned by mTORC1 and are specifically inhibited by the mTOR inhibitor, rapamycin (45), another study reported that the mTORC1 inhibitor, temsirolimus, increased the differentiation of mo-DCs (20). Given these findings, one may then consider that NOD2 might influence the differentiation of bone marrow precursors into tissue phagocytes in a context-dependent manner.

In this study, we provide experimental evidence that NOD2-dependent bacterial sensing by monocytes inhibits their differentiation in macrophages. Indeed, monocytes' fate can be influenced by the context. Such developmental switch occurs even in a context where their development from circulating monocytes is promoted into macrophages, upon activation of the metabolic signaling node mTORC1, which controls terminal differentiation of myeloid progenitors (46). We observed that this transition in the early steps of phenotypic developmental stages relied on the glycolytic-mediated control of monocytes/macrophages by the bacterial sensor NOD2 (47). We demonstrated that recognition of the gut microbiota by NOD2 is required for *de novo* reconstitution of mo-DCs that occupy the lamina propria of the murine intestine while having minimal effect on the mobilization of their precursors to the intestinal mucosa. Given that MDP is physiologically present in high concentrations within the intestinal lumen, our study set the stage to modulate NOD2-dependent signaling at the monocytic stage to avoid the activation of default developmental pathways, including mTORC1. These alternative developmental switches are probably leading to anti-TNF failure through an accumulation of inflammatory macrophages in the intestine of CD patients.

## Results

### Lack of NOD2 results in a competitive disadvantage for the mo-DCs pool within the colon and the peritoneal cavity in mice at steady-state that re-equilibrates during inflammation

**Figure 1:**
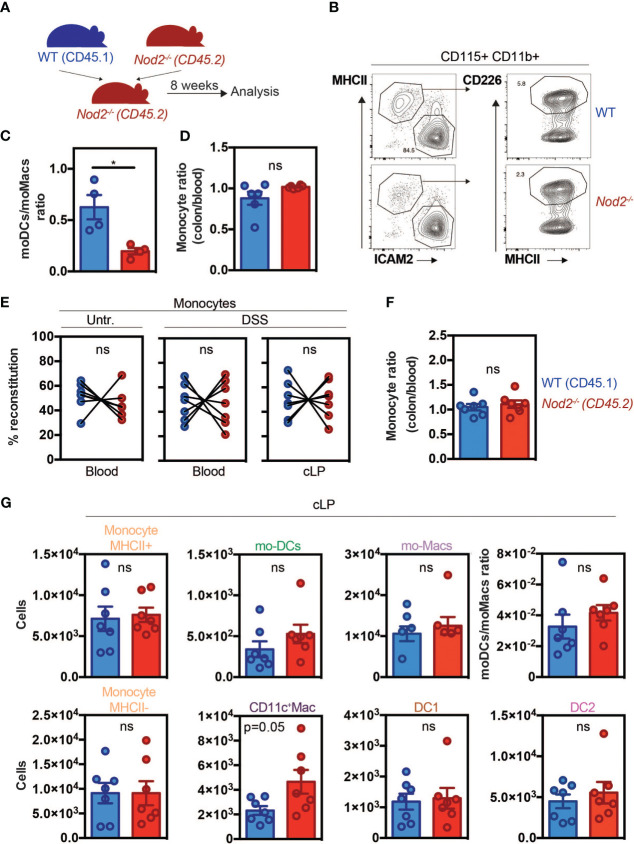
Nod2 signaling is required in BM cells to reconstitute the mo-DCs pool in the peritoneal cavity despite similar monocyte recruitment. **(A)** Experimental protocol. Mixed BM chimeras were generated by transferring WT (expressing CD45.1)(blue) and Nod2^-/-^ (expressing CD45.2) (red) cells in a 1:1 ratio into lethally irradiated Nod2-deficient recipients. Cells were isolated from the blood and the peritoneum 8 weeks after reconstitution. **(B)** Peritoneal cells were harvested and the proportion of mo-Mac (MHCII^-^ICAM2^+^) and mo-DCs (MHCII^+^CD226^+^) was determined by flow cytometry by gating within the CD115^+^CD11b^+^ cells. The frequency of the CD226^+^ DC is calculated within the CD115^+^ CD11b^+^ cells. **(C)** The ratio of mo-DCs/mo-Macs from frequencies obtained in B (n=4 mice). **(D)** Reconstitution index of Ly6C^+^ monocytes in the colon (CD11c^-^CD11b^+^Ly6C^+^CCR2^+^ gate) divided by the same ratio in the blood (n=6 mice). **(E)** 8 weeks after reconstitution, mixed chimera mice were treated with a 5-day course of 2% DSS (n=7 mice)**(E-G)**. **(E)** Monocytes content (in the blood: CD11b^+^ Ly6hi^+^, in the colon: CD11c^-^CD11b^+^Ly6C^+^CCR2^+^MHCII^-^) in the blood (CD11b^+^ Ly6hi^+^) of untreated and blood and colonic lamina propria cells (cLP) in DSS-treated mice. **(F)** Ratio of WT and Nod2-/- monocyte frequencies (colon/blood). **(G)** Colonic cells were analyzed as described in **Supplementary Figure 2A**. Numbers per million of monocytes MHCII^+^ (CD11c^-^CD11b^+^Ly6C^+^CCR2^+^MHCII^+^), mo-DCs (CD11c^+^CD11b^+^Ly6C^+^CCR2^+^MHCII^+^), mo-Macs (CD11c^-^CD11b^+^Ly6C^-^CCR2^-^MHCII^+^), monocytes MHCII^-^ (CD11c-CD11b^+^Ly6C^+^CCR2^+^MHCII^-^), CD11c^+^Macs (CD11c^+^CD11b^+^Ly6C^+^CCR2^-^MHCII^+^), DC1 (CD11c^+^CD11b^-^) and DC2 (CD11c^+^CD11b^+^Ly6C^-^CCR2^-^MHCII^+^) in the cLP (number of cells per million of live cells). The ratio of mo-DCs/mo-Mac from total cell number. Bars indicate mean ± SEM. Statistical significance was assessed by the non-parametric Mann-Whitney U test. ns, non significant.

Since Nod2 has been shown to regulate monocyte-derived cell differentiation in different contexts, we aimed to investigate whether in the colon this phenomenon is additionally a consequence of an impaired mobilization of monocytes from the BM as it was observed in C3H/HeJ Tlr4 mutant mice (7). Alternatively, maturation of Ly6C^high^ monocytes can follow different paths such as mo-DCs or CD11c^+^ macrophages which are considered as an intermediate between monocytes and macrophages (1). If NOD2 is intrinsically required for the development of Ly6C^high^ monocytes into mo-DCs in mice, inappropriate conversion of Ly6C^high^ monocytes into mo-DCs would be expected to promote the accumulation of macrophages. To this end, we realized mixed BM chimera mice. *Nod2*-deficient animals were lethally irradiated, and 24h later reconstituted with equal amounts (eg. 50:50) of BM cells from wild-type (WT) (CD45.1) and *Nod2*-deficient mice (CD45.2) (**Figure 1A**). CCL2 production and subsequent CD11b^+^Gr1^+^F4/80^+^ phagocytic cells’ recruitment to the colon have been shown to be reduced during *C. rodentium* infection in *Nod2^-/-^* mice (27), however, we did not expect a defect in monocyte recruitment in *Nod2^-/-^* mice at steady state. Peritoneal content of mo-DCs and mo-Macs within the peritoneum (8) and colonic tissue were analyzed 8 weeks after BM reconstitution as previously described (48) (**Figure 1**). Peritoneal cells express both CD115 and CD11b, they can be further subdivided into large MHC II^-^ CD102^+^ (ICAM2^+^) F4/80^+^ macrophages and MHC II^+^ CD226^+^ F4/80^lo^ subsets that differ in function and origin. The MHC II^+^ CD226^+^ F4/80^lo^ subset is Irf4-dependent and is continuously renewed by blood monocytes (49). These cells selectively express CD226, which is a marker of human mo-DCs, both *in vitro* and in ascites, and display a typical DC morphology profile (8). In our model, while the total numbers of mo-DCs and mo-Macs were increased in the *Nod2*-deficient competitive BM chimeras, no differences in frequencies were observed as compared to WT cells. However, within the peritoneum of these *Nod2*-deficient competitive BM chimeras, we observed a lowered relative proportion of mo-DCs when compared to mo-Macs in the CD45.2 cells as compared to the CD45.1 cells (**Figures 1B, C**; **Supplementary Figures 1A, B**). At steady-state, resident macrophage subsets have been shown to arise from Ly6C^hi^ monocytes in the intestine (10). In the colon, macrophages can be segregated into CD11c^+^CD11b^+^MHCII^+^Ly6C^+^ macrophages related to the CX3CR1^int^ inflammatory macrophages, and CD11c^-^CD11b^+^Ly6C^lo^MHCII^+^ monocyte-derived macrophages corresponding to the CX3CR1^hi^ tissue-resident macrophages (10, 50). Likewise what was observed in the peritoneal cavity, similar results were obtained in their colon in which the number of macrophages was significantly heightened (**Supplementary Figures 1A, B**) (**Supplementary Figures 2A, B** (gating strategy)). As *Nod2*-deficient mice are known to develop dysbiosis, we next assessed whether the capacity of blood monocytes to be recruited, to survive, and to expand into the colon may depend on the recognition of the gut microbiota by NOD2. Gut dysbiosis is responsible for decreased numbers of Ly6C^high^ monocytes in the spleen of mice at steady-state, however, MDP stimulation could restore splenic Ly6C^high^ cell frequency (51). In our settings, monocytes from either WT or *Nod2* ^-/-^ BM were similarly recruited in the colon of *Nod2*-deficient mice at steady-state (**Figure 1D**). We next assessed how inflammation may differentially alter the proportion of BM-derived phagocytes in the colon of *Nod2*-deficient chimeras. Colonic recruitment of CD11b^+^Ly6C^+^CD11c^-^MHCII^-^ monocytes has been shown to be a feature of murine colitis (52). While the segregation between CD11b^+^ DCs and macrophages could be arduous due to the appearance of a CD64-expressing population of mo-DCs, a subset of CD11c^+^ monocytes/macrophages has been shown to be involved in intestinal inflammation (39). Dextran sodium sulfate (DSS) was then administered in the drinking water of mixed-BM chimera mice to induce acute colitis (**Figures 1E–G**; **Supplementary Figure 2A, B** (gating strategy)). Such an established preclinical model of colitis is characterized by epithelial erosion, crypt loss, ulceration, and infiltration of immune cells. Similar reconstitution of WT and *Nod2^-/-^* Ly6C^high^ monocytes was observed in the blood and in the colon under DSS, as compared to untreated BM chimeras (**Figure 1E**). In agreement, the ratios of colonic and blood monocytes were equivalent between WT and *Nod2^-/-^* (**Figure 1F**). Likewise, the number of colonic Ly6C^high^ MHCII^+^ activated monocytes was not affected by Nod2 deficiency in response to inflammation (**Supplementary Figure 2C**). Furthermore, no difference in body weight loss was noticed at the time of the autopsy (data not shown). Upon inflammation, while the numbers of Ly6C^high^ monocytes, cDC, and the mo-DCs/mo-Macs ratio were equivalent in the colon of mixed BM chimera mice, CD11c-expressing macrophages were present in a greater number in *Nod2* ^-/-^ as compared to WT donor cells (p=0.05) (**Figure 1G**). These results show that NOD2 does not regulate the proportion of colonic mo-Macs at steady state *in vivo*, whereas it did alter mo-Mac and mo-DC ratios upon inflammation.

### Recognition of the gut microbiota by NOD2 regulates the reconstitution of intestinal mo-DCs from mobilized monocytes

**Figure 2:**
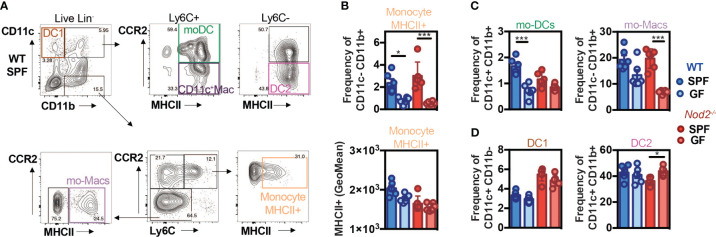
Default of conventional DCs and monocytes-derived cells recruitment in NOD2^-/-^ mice under microbiota deprivation. Colon Lamina Propria Mononuclear Cells (LPMC) frequency analysis in Wild-type (WT) (blue) and NOD2^-/-^ (red) mice raised under Specific-Pathogen Free (SPF)(sharp color) and Germ-Free (GF)(light color) conditions. **(A)** Gating strategy to determine the frequency of conventional DC1 (CD11c^+^CD11b^-^), of mo-DCs (CD11c^+^CD11b^+^Ly6C^+^CCR2^+^MHCII^+^), of CD11c^+^ Macs (CD11c^+^CD11b^+^Ly6C^+^CCR2^-^MHCII^+^), of conventional DC2 (CD11c^+^CD11b^+^Ly6C^-^CCR2^-^MHCII^+^), and the frequencies of mo-Macs (CD11c^-^CD11b^+^Ly6C^-^CCR2^-^MHCII^+^), and Mo^-^MHCII^+^ (CD11c^-^CD11b^+^Ly6C^+^CCR2^+^MHCII^+^), based on their CCR2, Ly6C and MHC levels, after exclusion of Lineage and doublet cells. **(B)** The frequency and MHCII GeoMean of CCR2^+^Ly6C^+^MHCII^+^ activated monocytes were evaluated in the monocyte-derived cells CD11c^-^CD11b^+^. **(C)** The frequency of mo-DCs was evaluated in the Ly6C^+^ cells. The frequency of CCR2^-^Ly6C^-^ mo-Macs was evaluated in the CD11c^-^CD11b^+^ monocyte-derived cells. **(D)** Frequency of DC1 cells (CD11c^+^CD11b^-^ gate) and DC2 cells (Ly6C^-^ gate). Bars indicate mean ± SEM (n=4-6/group). Statistical significance was assessed by two-way ANOVA. *P<0.05; ***P<0.001.

As the recognition of gut microbiota by NOD2 has been shown to be essential for the homeostasis of immune cells, we investigated more precisely whether it may impact the mononuclear phagocyte composition of the colonic lamina propria. The phagocytes composition was analyzed by flow cytometry in the colon of WT and *Nod2^-/-^* specific pathogen-free (SPF) and germ-free (GF) mice at steady-state using the markers CD11c, CD11b, CCR2, Ly6C, and MHCII (**Figure 2A**; **Supplementary Figure 3**). In the WT mice, the frequency of activated MHCII^+^ monocytes (**Figure 2B**) and of the proportion of mature tissue macrophages (**Figure 2C**) were reduced within the lamina propria of the colon of GF as compared to SPF WT mice, as observed in a series of studies (3, 53). By contrast, the expression level of MHCII on the classical subset of monocytes was similar within the colon of SPF *Nod2*-deficient mice as what was observed in the GF condition (**Figure 2B**, lower part). However, while the mo-DCs-like cells (CD11b^+^CD11c^+^Ly6C^+^CCR2^+^MHCII^+^) (54) proportions were decreased in GF WT mice as compared to SPF mice (**Figure 2C**), this difference was lost in *Nod2^-/-^* mice, highlighting the role of microbiota recognition and NOD2 in the accumulation of mo-DCs in the colon. As NOD2 has been shown to shape the recruitment of CD103^+^ DCs in the gut lamina propria (28), we reasoned that the loss of NOD2 signaling may have impaired either the trafficking or the development of some discrete subsets of cDC. To our surprise, only minor fluctuations were detected in the proportions of cDC1 and cDC2 that co-express or not CD11b respectively (**Figure 2D**). These results could be explained by the fact that cDC arise from a specific precursor. In addition, these cells were used as a control and we did not expect them to be affected in this context. Given that the gut microbiota does not regulate the abundance of cDC1, the decreased abundance of mo-DCs was not likely due to competition with cDC1 to occupy the colonic niche under homeostatic conditions. In addition, the moderate increase in cDC2 frequency observed in GF *Nod2* ^-/-^ mice was not observed in bone marrow chimera mice (**Figure 1**). This said, the number of CD11c-expressing macrophages was too low to conclusively apprehend potential differences in our experimental setting. Altogether, our data indicate that the Nod2-dependent recognition of the gut microbiota by monocytes when entering the colon from the blood is an important means by which NOD2 could facilitate the on-demand accumulation of intestinal mo-DCs from mobilized monocytes and subsequently may prevent the replenishment of macrophages. To exclude the potential influence of opportunistic pathobionts that may have been present in *Nod2*-deficient mice, the faecal microbiota from WT SPF mice was transplanted in GF recipients that are either deficient for NOD2 or not (**Supplementary Figure 4A**). The composition of mononuclear phagocytes was analyzed in the colon four weeks after transplantation such that their gut microbiota becomes similar to the one of the control mice (**Supplementary Figure 4B**). The frequency and absolute numbers of CD11c^+^MHCII^+^ and CD11c^-^MHCII^+^ mononuclear phagocytes were not significantly different in the GF *Nod2* ^-/-^ recipients as compared to GF WT recipients when exposed to WT microbiota (**Supplementary Figure 4B**). These results suggest that changes in the gut microbiota composition, that are pre-existing in the *Nod2*-deficient mice, could not be sufficient to influence the frequency of mononuclear phagocytes within the colonic lamina propria in mice. Altogether, our results indicate that recognition of the gut microbiota by Nod2 regulates the reconstitution of intestinal mo-DCs from mobilized monocytes.

### NOD2 signaling enhances the yield of mo-DCs from monocytes by inhibiting their differentiation in macrophages

**Figure 3:**
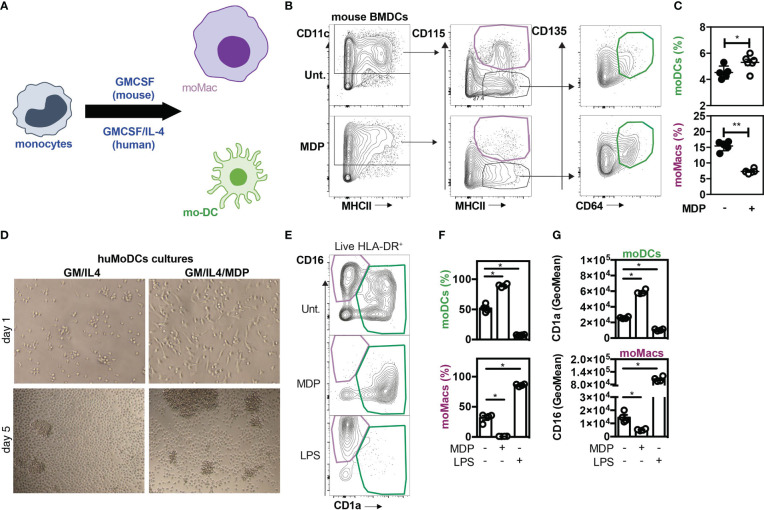
NOD2 stimulation is responsible for a mo-Macs/mo-DCs switch. **(A)** Experimental protocol. **(B)** Gating strategy for flow cytometry analysis of BMDCs upon MDP treatment. Mouse BM-derived cells were generated *in vitro* for 7 days in GM-CSF in the presence or not of MDP (10ug/ml). Mo-DCs were gated as CD11c^+^ MHCII^+^ CD115^-^ CD135^+^ CD64^+^ (green) and mo-Macs were gated as CD11c^+^ MHCII^+^ CD115^+^ (purple). Frequencies are calculated from the CD11c^+^ gate. **(C)** Frequencies of mo-DCs and mo-Macs in the mouse BMDC culture stimulated or not with MDP at the start of the culture. **(D)** Mo-DCs were generated by culturing CD14^+^ circulating human monocytes with GM-CSF and IL-4. MDP was added at the beginning of the 5 day-culture. Morphology of the differentiating cells at day 1 and day 5 of culture in the presence or not of MDP. **(E)** The impact of MDP or LPS stimulation at the start of the culture on mo-Macs (CD16^+^CD1a^-^) and mo-DCs (CD16^-^CD1a^+^) differentiation was evaluated by flow cytometry at day 6, after gating on live HLA^-^DR^+^ cells. **(F)** mo-DCs and mo-Macs frequencies. **(G)** CD1a and CD16 fluorescent mean intensity (GeoMeans) (n=5). These data are representative of at least 4 independent experiments with different mice **(B-C)** or donors **(D-G)**. Bars indicate mean ± SEM. Statistical significance was assessed by the non-parametric Mann-Whitney test. *P<0.05; **P<0.01.

In order to establish whether activation of NOD2 signaling may regulate mo-DCs development and therefore inhibit the differentiation of recruited monocytes toward macrophages, MDP was added at the start of the culture of mouse BM cells with a conditioning medium containing GM-CSF. When monocytes were cultured with murabutide, a NOD2 agonist, they exhibited enhanced TNF-α secretion as early as 6hours post-induction, which diminished at 24hours (9), suggesting that the effect of NOD2 stimulation is observable over a narrow window of time. The culture of mouse BM cells with the growth factor GM-CSF is a widely used protocol to generate either mo-Macs or mo-DCs within the CD11c^+^MHCII^+^ fraction (**Figure 3A**) (55). The percentage of CD115^+^CD64^+^ mo-Macs was significantly diminished while the frequency of mo-DCs significantly increased when BM cells were cultured in the presence of MDP as compared with the medium alone (**Figures 3B, C**). We then verified if similar results could be observed in *in vitro* culture of human monocytes. To this end, CD14^+^ monocytes were differentiated for 5 days in the presence of GM-CSF and interleukin-4 (IL-4), a well-established cocktail of conditioning soluble factors (56, 57), giving rise to both mo-DCs and mo-Macs (58). Light microscopy revealed that monocytes acquired an elongated shape as early as 24 hours after being exposed to MDP (**Figure 3D**). After 5 days of stimulation, we noticed the formation of a homotypic cluster of adherent cells with a probing morphology (**Figure 3D**). To support this, both adherent and non-adherent cells were harvested and stained at their surface for CD14, CD16, CD1a, and HLA-DR. Live and singlet cells were gated on HLA-DR-expressing cells and were systematically analyzed on day 6 (**Figures 3E–G**). Similarly to what is observed in *in vitro* cultures with M-CSF, TNF-α and IL-4 (9), early presence of MDP into such a culture system reduced the frequency of mo-Macs and their CD16 expression by five-fold (**Figures 3F, G**, lower part), while enhancing the yield of mo-DCs and their expression of CD1a (**Figure 3G**, upper part). Conversely, addition of LPS drastically diminished the frequency of mo-DCs in favor of mo-Macs that are expressing higher levels of CD16 (**Figures 3E–G**, lower part). By contrast, the yield of mo-Macs was similar between GM-CSF and IL-4 culture of monocytes that are treated or not with MDP for the last two days of culture (data not shown), suggesting an early involvement of NOD2 in shaping the development of mo-DCs in the disadvantage of mo-Macs *in vitro*. This observation is in agreement with the time-restricted ability of soluble factors such as IL-4 or TNF-α to induce the monocyte differentiation process toward mo-DCs in the first 72h of development (59, 60). This result is consistent with the steadily decline of NOD2 expression during the first 72h of the monocyte culture in the presence of GM-CSF and IL-4 (60). Indeed, monocytes differentiated for 72h with M-CSF, GM-CSF or GM-CSF^+^IL^-^4 have been described to exhibit transcriptomic, phenotypic, and functional divergences (60). While highly expressed in CD14^+^ monocytes, transcriptomic analysis determined that *NOD2* expression was maintained only by monocytes differentiated with GM-CSF but was rather decreased in monocytes differentiated with M-CSF and GM-CSF+IL-4. Accordingly, qRT-PCR analysis revealed a lowered expression of *NOD2* in terminally differentiated mo-DCs when compared to naïve monocytes (**Supplementary Figure 5**), indicating that the expression of *NOD2* is temporally regulated during monocyte differentiation. These results suggest that early NOD2 signaling conditions the differentiation of nascent phagocytes into mo-DCs that promote inflammatory responses. Consequently, those findings suggested that early NOD2 signaling may progressively promote the differentiation of rapidly mobilized monocytes into ontogenetically related cells with specific features of mo-DCs by inhibiting their conversion into mo-Macs within the lamina propria (61). Overall, our data indicate that MDP sensing by monocytes promotes *in vitro* their conversion into mo-DCs.

### NOD2 signaling acts through the mTORC1 pathway to license bifurcation of monocytes commitment

**Figure 4:**
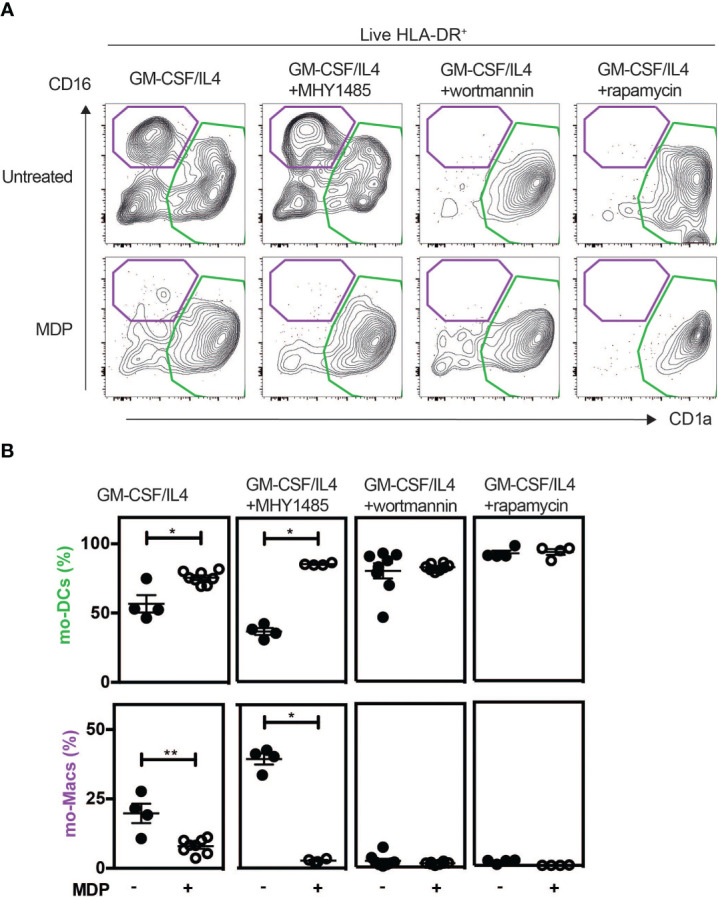
MDP enhances the differentiation of Mo-DCs in a mTORC1 independent manner. As described in **Figure 3D**, human CD14^+^ monocytes were treated or not with MDP, the mTOR activator MHY1485, the PI3K inhibitor wortmannin, or the mTOR inhibitor Rapamycin for 5 days at the same timepoint. Mo-DCs (CD1a^+^) and mo-Macs (CD16^+^) frequencies were assessed by flow cytometry using the HLA-DR, CD16 and CD1a markers. **(A)** Contour representing the mo-DCs (green) and mo-Macs (purple). **(B)** The frequency of mo-Macs and mo-DCs in the four conditions are depicted. Representative of 2 experiments with at least 3 biological replicates. Bars indicate mean ± SEM. Statistical significance was assessed by the non-parametric Mann-Whitney test. *P<0.05; **P<0.01.

We next asked how NOD2 signaling in monocytes modulates the unique property of monocytes to differentiate into macrophages or mo-DCs that are phenotypically and functionally different. To this end, we investigated the signaling events after NOD2 stimulation with a focus on the components of the mechanistic target of rapamycin (mTOR), mTORC1, and mTORC2, signaling pathways that are both involved in the generation and activity of tissue-resident peritoneal macrophages *in vivo (* 62, 63), and of human mo-Macs *in vitro (* 9). As we have previously shown with human monocytes (**Figures 3E, F**), the addition of MDP increased the yield of mo-DCs while decreasing the mo-Macs frequency (**Figures 4A, B**). However, the proportion of macrophages increased after the addition of MHY1485, which is a cell permeable activator that targets the ATP domain of mTORC1 but not mTORC2, used to promote the activation of mTORC1 (**Figures 4A, B**). These results highlighting the role of mTORC1 in the conversion of monocytes in macrophages are in agreement with what was observed with GM-CSF culture of mouse myeloid progenitors (64). Interestingly, MDP addition led to a significant decrease in macrophage frequency and an increase of mo-DCs yield, during the MHY1485 treatment, suggesting that NOD2 may act through mTORC1 during monocyte differentiation (**Figures 4A, B**). In line with our hypothesis, the proportion of mo-DCs was enhanced upon treatment of monocytes with wortmannin that acts upstream of the mTOR pathway similarly to what was observed with MDP alone (**Figures 4A, B**). Wortmannin is a selective inhibitor of phosphoinositide 3-kinases (PI3K), that can also block autophagy and has been described to irreversibly inhibit the serine-specific auto-kinase activity of mTOR (65). The PI3K/AKT pathway has been shown to negatively regulate the NOD2-mediated NF-κB pathway (66). As a result, the mo-Macs differentiation was completely inhibited. The addition of MDP to wortmannin, which is a non-specific, covalent inhibitor of PI3K that is also used for suppressing autophagy by interfering with autophagosome formation, did not increase mo-DCs frequencies. Cell toxicity was avoided as much as possible by treating cells for only 24 hours (data not shown). Similar results were obtained with rapamycin, the prototypic mTORC1 inhibitor, able to induce autophagy by potentiating LC3 lipidation (**Figures 4A, B**). These data are in agreement with the greater proportion of mo-DCs that is observed upon treatment with the mTORC1 inhibitor temsirolimus (20). These results suggest that NOD2 activation might negatively regulate the ability of the PI3K pathway to licensing the metabolic reprogramming of monocytes at an early stage of development.

### NOD2 activates mTORC2 pathway and promotes anaerobic glycolysis

In an effort to further understand how NOD2 activation may impair mTORC1-dependent macrophage differentiation, we cultured the human monocytic cell line THP1 that express NOD2 (67), which has been extensively used to study the development and function of human monocyte-derived phagocytes. THP1 cells were stimulated for 30 minutes with MDP, and the phosphorylation state of the Regulatory-associated protein of mTOR (also known as RAPTOR) at serine 792 (S792), which is mediated by AMP-activated protein kinase (AMPK), was quantified. This S792-phosphorylation of RAPTOR has been shown to reduce mTORC1 activity (68) and to act as a metabolic checkpoint that coordinates the energy status of each cell (68). In this experimental setting, activation of NOD2 signaling seemed to induce S792 phosphorylation of RAPTOR (**Supplementary Figure 6A**), suggesting that NOD2 could inhibit lipid uptake and foam cell formation through negative feedback on mTORC1 activity. At a time point of 24hours of stimulation, immunoblot analysis expectedly revealed that treatment with MDP did not increase the phosphorylation of S6K, which is a surrogate marker of mTORC1 activation (**Supplementary Figure 6B**). We next measured the phosphorylation of AKT at serine 473, as a surrogate marker of mTORC2 activation (64). In agreement with our hypothesis, we observed that a short treatment for 24 hours of THP1 cells with MDP increased this specific phosphorylation of the residue S473 (**Supplementary Figure 6B**). This phosphorylation was not observed in THP1 cells that lack the expression of NOD2. LPS also induced S473 phosphorylation, however, the phosphorylation of S473 was reduced when MDP treatment was followed by LPS stimulation (**Supplementary Figure 6B**). Consistent with that observation, MDP-treated THP1 monocytic cells were characterized by a NOD2-dependent upregulation of *IRF4* expression (**Supplementary Figure 6C**), which is a target gene of mTORC2 (69). Interestingly, LPS and Pam3 stimulations have been reported to decrease IRF4 expression in monocytes in a mTORC1-independent manner (9), suggesting that NOD2-mediated induction of IRF4 may be independent of this phenomenon. In line with the ability of NOD2 signaling to activate the Signal transducer and activator of transcription 5 (STAT5) (70), the treatment of THP1 cells with MDP significantly lowered the expression of IRF8 that is inhibited by STAT5 (71) (**Supplementary Figure 6C**). These results suggest that stimulation of monocytes with MDP induces mTORC2 activation and may act as a negative feedback loop on mTORC1, *via* the phosphorylation of AKTS473 and RAPTORS792 respectively. It has been postulated that GM-CSF may regulate *irf4* expression *via* STAT5 expression in monocytes and in macrophages (72). We could speculate that Nod2 may act in the same way and control IRF4 and IRF8 expression in monocytes *via* STAT5 activation. Metabolism changes, including in glycolysis and oxidative phosphorylation sustains energy needs of macrophages and dendritic cells, but also rewires their activation, polarization and differentiation as pro-inflammatory and anti-inflammatory cells (73–75). As the mTORC2 pathway plays a key role in the glycolytic reprogramming of monocytes that are rapidly mobilized on demand (76, 77), we next investigated if MDP treatment of monocytes is associated with changes in mTORC2-mediated metabolic cascade. To this end, previously published RNAseq data for MDP-treated vs untreated Ly6C^hi^ mouse monocytes (GEO accession number GSE101496) were mined for candidate genes encoding for enzymes involved in glycolysis. We observed an MDP-induced upregulation of several glycolytic genes such as the enzyme Gapdh that regulates the conversion of D-glyceraldehyde 3-phosphate into 1,3-bisphosphoglycerate (**Supplementary Figure 7A**) (26). To get further insights on how NOD2 may provide energy for monocytes, we next quantified the glycolytic capacity and reserve of THP1 cells that were deficient or not for NOD2. As THP1 cells mainly rely on glycolysis as a source of ATP for survival (78), these cells represent a suitable model. The oxygen consumption rate (OCR) was measured in NOD2-deficient and WT THP1 cells. We could observe that the lack of NOD2 could affect the OCR, a measurement of oxidative phosphorylation (OXPHOS) and extracellular acidification rate (ECAR), a surrogate measurement of glycolytic activity. Indeed, the extracellular acidification rate (ECAR) was measured by using a Seahorse bioanalyser. During the first step of glycolysis, we noticed a similar basal glycolytic rate between NOD2-deficient and parental THP1 cells (**Supplementary Figure 7B**). Upon blockade of oxidative phosphorylation of ADP to ATP by oligomycin, the basal glycolytic capacity of WT cells was similar to the one of THP1 *NOD2* ^-/-^ cells. By contrast, the inhibition of glycolytic H+ production by the competitive inhibitor of glycolysis, 2-Deoxy-D-glucose (2-DG), revealed a trend to a lower glycolytic reserve in the absence of NOD2. These results suggest that NOD2 may promote a lower pH, by sustaining a higher glycolytic demand, which is required for mo-DCs differentiation (20). TNF-α or IFNγ stimulations have been shown to induce a metabolic reprogramming of macrophages toward a pro-inflammatory M1 phenotype (79). NOD2 triggering may favor a switch in glycolysis to influence monocyte differentiation *via* cytokine production. Altogether, these results indicate that NOD2 signaling of monocytes may interfere with the PI3K/mTORC1 pathway through the mTORC2/AKT complex, which might inhibit the diversion of monocyte differentiation to macrophages *via* metabolic reprogramming (76). In other words, these data suggest that MDP may induce a transient negative regulation of the mTOR pathway to limit the accumulation of macrophages, leading to an unrepressed generation of mo-DCs.

### The inhibition of the mTORC1 pathway by NOD2 promotes the secretion of TNF-α

**Figure 5:**
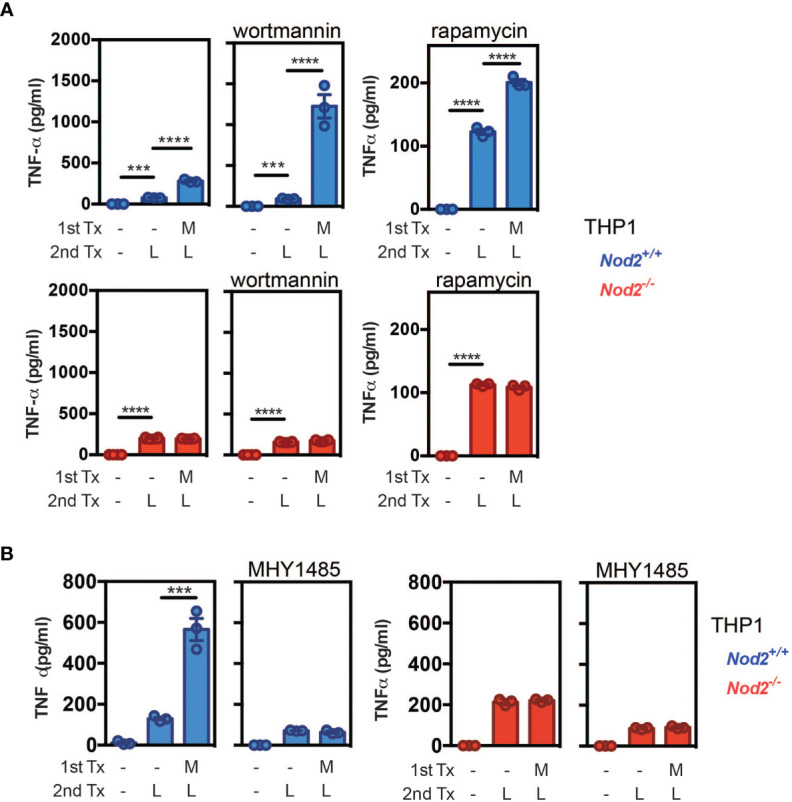
NOD2 signaling is hierarchically dominant over mTORC1 activation to condition monocyte differentiation into mo-DCs. **(A)** THP1 (blue) and THP1 *NOD2*-deficient cells (red) were treated as described in the material and method section to evaluate the LPS responsiveness of MDP-treated cells. In addition, the PI3K inhibitor wortmannin or the mTOR inhibitor rapamycin were added or not for 24h. TNF-α production was measured by ELISA. **(B)** LPS responsiveness was evaluated by measuring TNF-α production in THP1 WT or *Nod*2^-/-^ cells treated with MDP, MHY1485, or both for 24h. Bars indicate mean ± SEM at least three biological replicates and data are representative of 2 independent experiments. Statistical significance was assessed by ordinary one-way multiple comparisons. ***P<0.005 ****P<0.001.

We previously observed that early MDP treatment can condition and activate the metabolic pathways of monocyte-derived cells and also promote the differentiation of mo-DCs to the detriment of mo-Macs. Since bacteria are able to stimulate multiple PRRs, and chronic stimulation with MDP has been shown to down-regulate TLR4-induced TNF-α secretion by human monocyte-derived macrophages (80), we next experimentally addressed the functional impact of early NOD2 signaling on the ability of nascent phagocytes to respond to LPS when synergizing with MDP (81). Of note, while a short pre-incubation with MDP has been shown to act synergistically with LPS to induce the synthesis of TNF-α in monocytes (82), a decrease of TNF-α secretion and other pro-inflammatory cytokines, referred to as tolerance to TLR4 restimulation, appears after 24hours of pre-treatment with MDP in human monocyte-derived macrophages (80). Furthermore, mice injected with MDP were protected against TNBS- or DSS-induced colitis by the suppression of multiple TLR pathways (29) suggesting a cross-tolerization of TLRs by chronic NOD2 stimulation (83). To evaluate the LPS responsiveness of MDP-treated cells, THP1 and THP1 cells that do not express NOD2 (THP1 *NOD2^-/-^*) were incubated sequentially with MDP and then LPS or LPS alone. As expected, the cells pre-treated with MDP enhanced their subsequent cytokine response upon LPS treatment (**Figure 5A**, left upper part). This synergistic effect on the production of TNF-α to subsequent treatment with LPS was blunted with THP1 *NOD2^-/-^* (**Figure 5A**, lower part). In order to confirm our data with primary cells, mouse BM monocytes were cultured in the presence of MDP. The responsiveness to LPS was next analyzed by measuring the release of Tnf-α by specific ELISA. An apparent synergistic effect on the secretion of Tnf-α was retained in those cells that were primed with MDP (**Supplementary Figure 8A**). This synergistic effect on the production of Tnf-α to subsequent treatment with LPS was absent in monocytes that were isolated from the BM of *Nod2*-deficient mice (**Supplementary Figure 8B**). Such a functional model of hierarchy could be of particular importance in the context of loss of bacterial tolerance or the need for the replenishment of tissue mo-DCs following injury or infection. We then hypothesized that activation of NOD2 signaling may influence the bioenergetic needs of nascent mo-DCs by interfering with the mTOR pathway. In agreement with the autocrine role of NOD2-mediated TNF-α secretion on the development of mo-DCs (9), the PI3K inhibitor wortmannin increased by 3-fold the secretion of TNF-α upon stimulation of MDP-primed THP1 cells with LPS when compared to control cells (**Figure 5A**, blue, upper part). This effect of wortmannin on the monocyte responsiveness to LPS is lost with MDP-primed THP1 cells that do not express NOD2 (**Figure 5A**, red, lower part). In agreement with what was observed with wortmannin, rapamycin treatment, which inhibits mTOR, did not impair the secretion of TNF-α by THP1 cells when stimulated with MDP (**Figure 5A**). On another hand, bafilomycin treatment, which inhibits autophagy at the final step of fusion of lysosome with autophagosome, blunted the synergistic effect of MDP (data not shown). Interestingly, we found that the activation of mTORC1 with MHY1485, which is supposed to promote a macrophage phenotype (**Figure 4B**), inhibited the MDP-induced secretion of TNF-α by THP1 cells (**Figure 5B**). However, no TNF-α secretion was observed in *NOD2*-deficient THP1 even in the presence of MHY1485, suggesting that in our settings, MDP was not able to impair the effect of the macrophage prone drug MHY1485 on TNF-α production. Altogether, these results suggest that MDP, in synergy with LPS, can induce TNF-α secretion that may influence the differentiation of monocyte-derived cells toward mo-DCs. Given the effect of mTOR pathway inhibition on TNF-α secretion, we can speculate that NOD2 signaling influences monocyte fate decision over the activation of mTORC1 for conditioning their differentiation into mo-DCs *via* TNF-α induction.

### NOD2 loss-of-function mutation impairs the phenotypic switch of monocytes in CD patients in a TNF-α dependent manner

**Figure 6:**
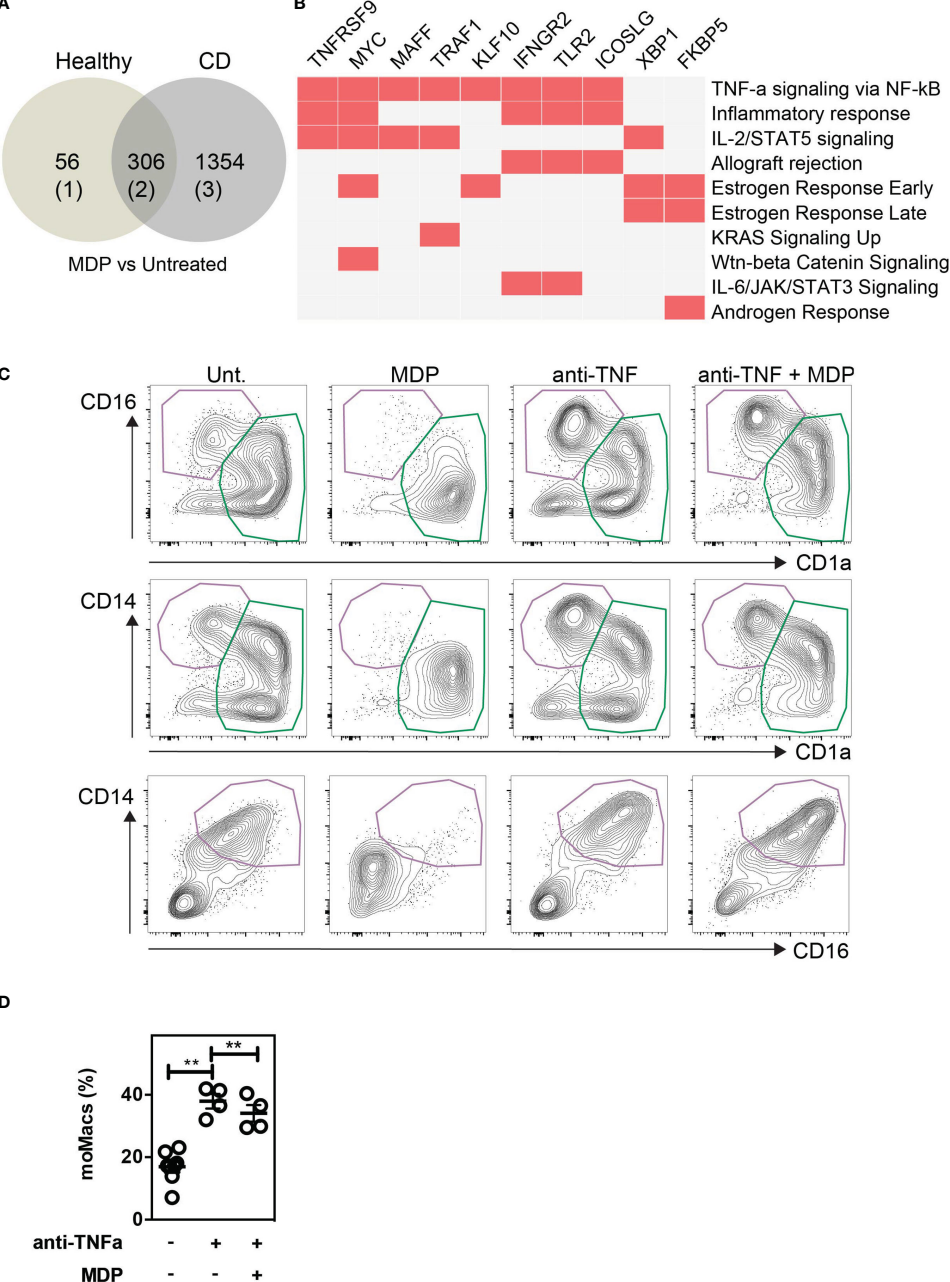
NOD2-induced decrease of macrophages is dependent on TNF-α production. Public RNA-seq data set of untreated or MDP-stimulated monocytes isolated from peripheral blood of healthy controls (n=2) and CD patients in complete remission were analyzed. Among the CD patients, 3 are bearing and 2 are not bearing the loss of function mutation in the *NOD2* gene. **(A)** Venn diagram comparing the Differentially Expressed Genes (DEG) between MDP-treated and untreated in healthy controls and CD patients. **(B)** Geneset enrichment analysis (GSEA) among the 156 commonly differentially upregulated genes that are induced by MDP in CD14-expressing cells from either control or CD patients. **(C)** Monocytes were treated or not with MDP, anti-TNFα (Adalimumab), or both during GM/IL4 cultures. Isotype was used as a control. Mo-DCs and mo-Macs frequencies were assessed by flow cytometry using the CD14, CD16 and CD1a markers on day 6 of the culture. Contour plot representing the mo-DCs (green) and mo-Macs (purple). Representative of at least 2 independent experiments with different donors and with at least 4 biological replicates. **(D)** The frequency of mo-Macs upon anti-TNFα and MDP treatment is depicted. Bars indicate mean ± SEM. Statistical significance was assessed by the non-parametric Mann-Whitney. **, P<0.001.

To evaluate the importance of a monocyte phenotypic switch in CD patients with NOD2 mutations, we used a published RNA-seq data set from an exploratory cohort that is deposited in the GEO database (GSE69446) (84). Given that CD14-expressing cells are obligate precursors of discrete subsets of phagocytes that play a role in CD pathogenesis, the monocytes were isolated from peripheral blood of healthy controls (n=2) and CD patients in complete remission for at least 4 weeks prior to inclusion. Among those five patients, three carried the loss-of-function mutation in the *NOD2* gene, referred to as 1007fs mutation. As depicted in the Venn-diagram (**Figure 6A**), MDP treatment of monocytes from healthy donors and CD patients significantly modified the expression of up to 362 and 1,660 genes, respectively. Among those, a list of 306 genes was commonly regulated by MDP in blood monocytes from both control and CD patients. Geneset enrichment analysis (GSEA) indicated that the pathway “TNF-alpha signaling *via* NF-kB” (24/200)(adjusted p-value 3.12E-20) was significantly induced among the 156 commonly differentially upregulated genes that are induced by MDP in CD14-expressing cells from either control or CD patients (**Figure 6B**; **Supplementary Data Set 1**). In addition, the “mTORC1 signaling” pathway (4/200)(adjusted p-value 0.13) tended to be upregulated by MDP. The pathway “Wnt-beta Catenin Signalling” (3/42)(adjusted p-value 0.019) was also significantly induced among the 156 common genes induced by MDP in control and CD cells (**Supplementary Data Set 1**). Moreover, we noticed an enrichment of up-regulated genes related to dendritic cells (9/199)(adjusted p-value 7.8E-3) (**Supplementary Data Set 2**), among those *NR4A3* is involved in the proper differentiation of Mo-DCs (85). Furthermore, a list of down-regulated genes that are related to diverse subsets of tissue macrophages was identified by using GSEA (11/204)(adjusted p-value 1.1E-5) (**Supplementary Data Set 3**). Among the CD patients, we next assessed whether cells bearing an unfunctional NOD2 (n=3), as homozygous for the 1007fs NOD2 mutation, may differentially respond to MDP. This led us to identify a specific loss of MDP-induced expression of 10 genes, including *IL12B* and *miR-155* in mutated patients as compared to the patients who did not bear this mutation (n=2) (**Supplementary Data Set 4**). Inhibition of miR-155 in human monocytes by an antagomir during 6h increased significantly MAFB (9), a transcription factor implicated in the molecular control of monocyte-macrophage differentiation (86). Pathway enrichment analysis identified a set of genes among the 17 that were implicated in TNF-α signaling *via* NF-kB (5/200)(adjusted p-value 2.53E-7) (**Supplementary Data Set 5**) and TNF-α effects on cytokine activity, cell motility, and apoptosis (4/135) (adjusted p-value 1.75E-4) (**Supplementary Data Set 6**). Accordingly, the Kyoto Encyclopedia of Genes and Genomes (KEGG) database analysis of the 941 up-regulated genes in CD cells which are not present in control cells after MDP treatment identified a set of genes related to “TNF signaling pathway” (28/112)(adjusted p-value 5.6E-11) (**Supplementary Data Set 7**). These results extracted from transcriptomic data suggest that MDP might regulate and affect the differentiation of monocytes into the macrophage pathway by the induction of DC-inducing soluble factors, such as TNF-α. We then evaluated whether the formation of mo-DCs, which is initiated by NOD2 signaling, is inhibited upon neutralization of TNF-α with adalimumab, which is a fully human anti-TNF-α monoclonal antibody. Interestingly, it has been shown that the percentage of classical monocytes was higher in patients responding to adalimumab than in patients not responding to the same drug (87). CD14-expressing cells have been cultured for 5 days in a medium with GM-CSF, IL-4, MDP, and/or adalimumab (**Figures 6C, D**) and/or isotype control (data not shown). In contrast with the isotype control, the addition of adalimumab in the medium together with GM-CSF and IL-4 significantly increased the frequency of mo-Macs (**Figures 6C, D**). Among those, it has been noticed that some co-express the macrophage marker CD14 (88). Interestingly, in the presence of MDP, mo-DCs did not express the marker CD14 (**Figure 6C**). Monocytes in the presence of MDP produced higher levels of TNF-α (**Supplementary Figure 9**). In conclusion, NOD2 may molecularly define an education process that subsequently prevents the accumulation of monocyte-derived macrophages.

## Discussion

We report herein experimental evidence that monocytes fail to differentiate into mo-Macs when the NOD2-mediated signaling is activated. By using competitive BM chimera, we did not observe a lack of recruitment of Ly6C^hi^ monocytes in the colon, suggesting that systemic MDP does not affect the number of recruited colonic monocytic cells at baseline, but may rather trigger early changes in epigenetic regulation of mo-DCs development (89). Besides the Nod2-dependent regulation of GM-CSF secretion by stromal cells in mice (28), our data highlighted a Nod2-dependent regulation of a developmental process of mo-DCs by the gut microbiota that is likely solicited when the DCs population must be replenished after fecal transplantation. This is particularly true within the first years of life, in which the immunological tolerance is not yet fully operational. One may anticipate that such demand-driven generation of mo-DCs is likely dependent on several mechanisms governing tolerance to MDP that are programmed in time for keeping a fine balance between each discrete subsets of phagocytes with context-dependent functions. Overall, our data suggest that MDP sensing by monocytes could promote *in vivo* their early conversion into mo-DCs for the maintenance of intestinal homeostasis at the expense of inflammatory macrophages.

Chronic stimulation of monocytes with MDP causes what is often called NOD2-induced tolerance which consists of tolerance to MDP and other bacterial signals such as LPS (90). On another hand, MDP can restore cytokine production, including TNF-α production, in LPS-tolerized macrophages (91). Proteosomal degradation of NOD2 protein confers rapid induction of refractoriness to MDP that protects the host from tissue damage or even death (90, 92). Such negative feedback regulatory mechanism fails to occur when human primary monocytes or transfected cell lines are defective in either the E3 Ubiquitin ligase ZNRF4 or the protein NLRP12 (48, 93). Consequently, treatment with MDP of monocyte-derived cells that are deficient for the aforementioned molecules led to an excessive inflammation with a sustained NF-κB activation. We have observed NOD2-mediated induction of IRF4. A proteo-analysis of HEK response to MDP highlighted that the Crk-like protein and the phosphoglycerate kinase 1 (PGK1) are upregulated in the NOD2-WT as compared to controls. Crk-like protein is an oncogene and an adaptor protein that has been shown to associate with STAT5, while PGK1 protein has been shown to be an enzyme of the glycolytic pathway (94). According to the ability of NOD2 signaling to activate STAT5, we observed a decrease in IRF8 expression with MDP (**Supplementary Figure 6C**).

One may infer that blood monocytes deficient for Nod2 may develop into inflammatory macrophages in the gut and lead to an increased inflammatory response in colitis for instance. Different studies have shown that most of the immune cells, particularly myeloid cells, may actually have dual activity, pro-inflammatory or immunosuppressive, depending on the signals received from the microenvironment (95). As this dual display of antagonizing functions exists also in conventional dendritic cells (96), we anticipated that similar dual properties of mo-DCs would likely exist and be influenced by microbial-derived products in their microenvironment.

The notion that metabolic control is upstream of inflammatory function has been proposed recently (97). Indeed, circulating monocytes and monocyte-derived macrophages from patients with fibroinflammatory vasculopathy are highly efficient in glucose import and are expressing higher glycolysis-associated genes (GLUT1, HK2, PKM2, LDH, c-myc, and HIF-1α) in comparison to healthy individuals. Consistently, the expression of the Aldoa and Aldoc that are converting F1,6BP into GADP and of the Gadph that is converting GADP into 1,3BPG were significantly upregulated in Ly6C^hi^ mouse monocytes that were treated by MDP as compared to untreated (**Supplementary Figure 7A**) (26). Similarly, MDP stimulation enhanced the expression of the Pgk1 converting 1,3BPG into 3-P-G and the Pagm1 that is involved in the conversion of the latter into 2PG. Aside from these enzymes, the Ldhb which is converting the Lactate into Pyruvate was downregulated, as well as the HK-II which is converting the glucose into glucose 6-phosphate (G6P). In addition, to confirm in human the MDP-mediated metabolic switch toward glycolysis observed in mouse monocytes, we analyzed the RNA-seq data set from an exploratory cohort of CD patients carrying a loss-of-function mutation in the *NOD2* gene and in complete remission vs healthy controls (as described in **Figure 6B**). Among the common 156 up-regulated genes induced by MDP either in control and CD monocytes as compared to untreated, analysis of MSigDB database indicated also the pathway “mTORC1 signaling” and containing SLC7A5, XBP1, HSPA5, PPA1, AK4, and CFP (**Supplementary Data Set 1**). Further studies will be needed to better understand the MDP-induced regulation of glycolysis enzymes and their regulation of mTORC1 signaling (98). Additionally, among the genes having a loss of MDP-induced expression in monocytes from SNP13 CD patients, *AK4* is involved in the positive regulation of mouse myeloid cells glycolysis and inflammatory cytokine production such as Tnf-α and Il-6 (99). In addition, we observed a decline of oxidative phosphorylation and glycolytic activity in NOD2-deficient THP1 cells suggesting a metabolic switch after NOD2 stimulation. As the induction of metabolic enzymes seems to appear rapidly after MDP stimulation (**Supplementary Figure 7**), glycolysis may precede the production of inflammatory cytokines during mo-DCs/mo-Macs differentiation (97). MDP has been shown to induce a metabolic reprogramming of human Monocyte-derived Macrophages (MDM) and a lower level of glycolysis than MDM, but comparable OXPHOS in MoDC at basal conditions (44). Moreover, a NOD2 agonist injected *in vivo* increased both glucose consumption and lactate release in mouse peritoneal macrophages and increased TNF and IL-6 production. While the effect of the addition of MDP can be detected very rapidly on glycolysis and OXPHOS (e.g. 10min), cytokine production can be detected after 3h of culture of human mo-Macs or mo-DCs (44). In addition, it has been suggested that the increase of glycolysis may take place without *de novo* gene synthesis in MDM upon treatment with NOD1 or NOD2 agonists due to the fact that it occurs after 1h of stimulation (44). However, while IRF5 has been shown to be necessary for NOD2-induced glycolysis, TNF-α, IL-1β or IL-12 could promote glycolytic gene expression and glycolysis in an IRF5-dependent manner in macrophages (47). Moreover, the autocrine pro-inflammatory cytokines were required for glycolysis suggesting a positive regulation of TNF-α and IL-1β on NOD2-mediated glycolysis in polarized and unpolarized macrophages. Of note, the knock-down of glycolytic genes induced the decrease of cytokine release, highlighting the double feedback regulation of cytokines and glycolytic genes. It has been mentioned that 2-DG partially prevented mo-DC differentiation, without affecting cell viability (20). Moreover, 2-DG appeared to block NOD1 agonist– or LPS-induced elevation of ECAR in mo-Macs and to inhibit LPS-induced TNF-α, IL-6, and IL-12 production by mo-DCs (44). Treatment with 2-DG has also been shown to inhibit Flt3L-induced proliferation of mouse precursors in a dose-dependent manner, indicating that aerobic glycolysis is involved in DC development (75). The NOD2/Ataxin-3 axis has been described in the regulation of myeloid cell metabolism (100). Indeed, ataxin-3 depletion led to a significant reduction in the oxidative phosphorylation of THP1. 2DG treatment has also been involved in the decrease of TNF, IL-1β, and IL-10 secretion and the reduction of the expression of genes involved in innate immune signaling pathways, cytokines secretion, and ROS production in LPS-stimulated human monocytes (101) suggesting a rewiring of monocyte function after glycolysis blockade. In addition, 2-DG treatment increased IL-23 secretion in GM-DCs *in vitro* and *in vivo* after imiquimod stimulation and promoted an increase of Ddit3 and Xbp1s expression in imiquimod-treated GM-DCs (102). XBP1s, a transcription factor that has been associated with CD (103), is proposed to play a major role in the development, differentiation, survival, and immune responses of various immune cells, including dendritic cells and macrophages (104), suggesting that glycolysis inhibition by 2-DG could affect monocyte-derived cell differentiation after PRR activation. As microbiota-derived circulating peptidoglycan is found in the mouse blood (105), one can propose that MDP can induce a metabolic reprogramming of circulating monocytes leading to a higher glucose consumption which regulates their mitochondrial activity, such as ROS production and effector molecules expression such as TNF-α. Here, we demonstrated that blocking TNF-α with adalimumab, which has been described to bind to membrane TNF-α with relatively higher affinity than etanercept (106), limits the development of MDP-induced mo-DCs. Similarly to our observations with the *in vitro* generated human mo-DCs with GM-CSF and IL-4, it was recently shown that MDP stimulation of human monocytes in *in vitro* culture with IL-4, M-CSF, and TNF-α promotes the generation of mo-DCs and limits the one of macrophages. In addition, intradermal injection of TNF-α into the ear of mice increases mo-DC numbers (9).

Interestingly, DC obtained from either *Tnfr1* ^−/−^ mice or patients treated with anti-TNF-α showed an unusual mixed immature/mature phenotype (107, 108) suggesting the development of macrophages in these cultures (55, 109). While TNF-α is a weak stimulator of CCR7 expression (110), it counterbalances the emergence of M2-like tumor macrophages (111). Such cytokine is sharply induced by microbiota, in Nod2-dependent and –independent pathways, during weaning for lowering the risk of developing colorectal cancer later in life (112). Conversely, an impaired dendritic cell function has been reported in most CD patients with NOD2 1007fs mutation (113). While TNF-α can upregulate NOD2 expression in myelomonocytic cell lines (67), anti-TNF therapy could alter the Nod2-induced equilibrium between discrete subsets of intestinal phagocytes with different properties. Additional analysis of published RNAseq data of the CD patients’ cohort in remission and healthy controls, while comparing to untreated, indicated that the pathway “Wnt-beta Catenin Signalling” was also significantly induced among the 156 common genes induced by MDP either in CD14-expressing control and CD cells including MYC, HEY1, JAG1 and A Disintegrin And Metalloproteinase 17 (ADAM17) (**Supplementary Data Set 1**). Wnt-beta catenin signaling pathway limits the differentiation into macrophages of BM cells cultured with GM-CSF (69). It is worth noting that A Disintegrin And Metalloproteinase 17 (ADAM17 (also known as TNF-alpha converting enzyme (TACE)) is a sheddase with a broad range of substrates such as membrane-bound TNF-α (114) and M-CSF receptor (59). ADAM17-dependent cleavage of M-CSF receptor is the mechanism by which GM-CSF and IL-4 block M-CSF- and RANKL-induced osteoclast differentiation from monocytes (59). Modulating the NOD2/TNF-α signaling axis to balance the induction of mo-DCs and repression of mo-Macs appears to be a promising new target for immunotherapy of colorectal cancer and to treat stricturing complications of CD patients.

Additionally, among the 17 genes losing MDP-induced expression in monocytes from CD patients with the homozygous SNP13 mutation, *NR4A3* is involved in the proper differentiation of Mo-DCs (FC 1.72, adjusted p-value 0.00206) (**Supplementary Data Set 4**) (85). One may suggest that inhibition of M-CSF receptor signaling by MDP on monocytes is required to impair macrophage differentiation in certain circumstances. For instance, type 1 cysteinyl leukotriene receptor (CYSLTR1) was significantly down-regulated from healthy controls or CD patients under MDP treatment (84). Inhibition of CYSLTR1 prevents M-CSF- and RANKL-induced osteoclast differentiation of BM precursors (115). Additionally, the ETS variant transcription factor 3 (ETV3) was significantly up-regulated within the MDP-treated monocytes from healthy controls or CD patients. It is induced by the anti-inflammatory cytokine IL-10 (116), and blocks M-CSF-induced macrophage proliferation (117). In CD patients, a unique response to MDP was observed with 941 and 413 up-regulated and down-regulated genes respectively (84). Analysis of Azimuth Cell Type database showed enrichment of up-regulated genes that are related to “Myeloid Dendritic Type 1” among which CCR7 is a hallmark of DC as it is critically required for their migration to lymph nodes (118). Equally of importance, the transcription factor *NRA43*, which is involved in the proper differentiation of mo-DCs (85), is less induced by MDP treatment in monocytes from CD patients than in cells from healthy controls (FC 1.6 and FC 1.71, respectively) as compared to untreated.

Thus, by using BM chimera and fecal transplantation models, our study highlights the role of NOD2 and the microbiota in the reconstitution of monocyte-derived cells in the colon of mice at steady state while not affecting mo-Macs during colitis. NOD2 promotes mo-DCs development by interfering with mouse BM and human monocyte differentiation in macrophages. In addition, we observed that NOD2 impacts mo-DCs/mo-Macs differentiation through activation of the mTORC2 pathway and a metabolic switch in NOD2 transfected THP1 involving TNF-α. The involvement of TNF-α signaling pathway has been confirmed in transcriptomic data of CD patients. Here, we provided a better understanding of macrophage-differentiation inhibition by NOD2-mediated signaling, which may offer new therapeutic strategies aiming at limiting the detrimental effects of pathogenic macrophages in gut pathologies such as CD.

## Materials and methods

### Mice

All animal studies were approved by the local investigational review board of the Institut Pasteur of Lille (N°28010-2016012820187595). Animal experiments were performed in an accredited establishment (N° B59-108) according to governmental guidelines N°86/609/CEE. Age-matched and gender-matched C57BL/6J WT, Nod2-deficient mice (Nod2^-/-^), have free access to a standard laboratory chow diet in a temperature-controlled SPF environment and a half-daylight cycle exposure. C57BL/6J WT GF, Nod2^-/-^ GF mice were bred at TAAM-CNRS and were transferred into autoclaved sterile micro-isolator cages. C57BL/6J WT mice were purchased from Janvier Laboratories, France. Ly5.1 WT mice (CD45.1) were purchased from Charles Rivers Laboratories, France. *Nod2^–/–^* mice were provided by R.A. Flavell (Yale University School of Medicine, Howard Hughes Medical Institute).

### Induction of acute colitis

A single cycle of acute colitis was induced by giving mice 2% (wt/vol) DSS (TdB Consultancy) for a period of 4 to 5 days followed by normal drinking water for the indicated period of time with a threshold of the maximal lost weight of 20% of the initial weight. DSS was dissolved in drinking water and changed every 3 days. Signs of morbidity, including body weight, stool consistency, occult blood, or the presence of macroscopic rectal bleeding, were checked daily. At specific time points throughout the course of the challenge, mice were autopsied to assess the severity of the disease by measurement of colon lengths and cell composition by flow cytometry.

### Bone marrow transplantation experiments

Recipient mice underwent a lethal total-body irradiation (2X 5.5Gy, 4h between each dose). Twenty-four hours post-irradiation, mice received intravenously 2 × 10^6^ fresh BM cells. *Nod2*-deficient animals were irradiated and reconstituted in a 1:1 ratio with bone marrow cells from WT (CD45.1) and *Nod2*-deficient mice (CD45.2). Blood was collected in heparin-containing tubes 7–8 weeks after BM transplantation and reconstitution efficiency was checked by flow cytometry (119). Cellular content within the colon, the blood, and the peritoneum of chimeric mice were analyzed 8 weeks after BM reconstitution by flow cytometry. In some experiments, DSS was administered for 5 days in the drinking water of mixed-BM chimera mice to induce acute colitis.

### Fecal transplantation

Fecal microbiota from WT mice was transplanted by gavage in GF mice that are deficient or not for Nod2 (120). The mice were used after four weeks of colonization with feces from WT mice.

### Isolation of mouse colonic lamina propria cells

Lamina Propria Mononuclear Cells (LPMC) were prepared from murine intestines by enzymatic digestion as previously described (3). Briefly, cells were isolated from colons, after removal of epithelial cells, by enzymatic digestion with 1.25 mg/ml collagenase D (Roche Diagnostics), 0.85 mg/ml collagenase V (Sigma-Aldrich), 1 mg/ml dispase (Life Technologies), and 30 U/ml DNaseI (Roche Diagnostics) in complete RPMI 1640 for 30–40 min in a shaking incubator until complete digestion of the tissue. After isolation, cells were passed through a 40μm cell strainer before use (BD biosciences). Colonic cell numbers were determined by counting beads and following the manufacturer’s instructions (AccuCheck counting beads, Invitrogen).

### Generation of bone marrow-derived dendritic cells

Bone marrow cells were flushed out of the mouse bones with complete RPMI 1640 (Gibco). A single-cell suspension was then prepared by repeated pipetting. Bone marrow-derived dendritic cells (BMDCs) were generated for 7 days in respectively RPMI-1640 medium (Gibco), supplemented with glutamine, penicillin, streptomycin, 2-mercaptoethanol (all from Gibco), and 10% heat-inactivated fetal calf serum (GE Healthcare). The medium was supplemented with 20 ng/ml GM-CSF of J558 cells (GM-CSF-producing cells). Half of the medium was removed at day 3 and new medium supplemented with GM-CSF supernatant (2x, 40 ng/ml) was added (55). When mentioned, muramyl dipeptide (MDP) (10μg/ml; Invitrogen) was added from the beginning of a 6-day culture with GM-CSF (day 0).

### Human monocyte-derived dendritic cell generation

Human Peripheral Blood Mononuclear Cells were prepared from buffy coats (Etablissement Francais du Sang (EFS), Lille, France) using Ficoll Paque (Lymphoprep, StemCell). The use of human samples was approved by the French Ministry of Education and Research under the agreement DC 2013-2575. According to French Public Health Law (art L 1121–1-1, art L 1121–1-2), Institutional Review Board and written consent approval are not required for human non-interventional studies. Monocytes were positively isolated using CD14^+^ microbeads (Miltenyi Biotec) according to the manufacturer’s recommendations. Cells were cultured for 6 days with rhGM-CSF (20ng/ml; Peprotech) and rhIL-4 (5ng/ml; Peprotech). When mentioned, muramyl dipeptide (MDP) (10μg/ml; Invitrogen) was added from the beginning of a 5-day culture with GM-CSF and IL-4 (day 0). The mTOR activator MYH1485 (2μM, Sigma), wortmannin (1μM, Sigma) or rapamycin (100nM, Sigma) were added at the start of the culture (day 0). Adalimumab (Humira M02‐497) was a gift from Abbott (Abbott Park, IL, USA).

### Cytokine measurement

Cytokine levels were determined by ELISA kits (DuoSet), according to protocols provided by R&D Systems.

### Western blot

Protein extraction was performed using RIPA buffer in the presence of a complete Mini EDTA-free protease inhibitor (Roche) and PhosSTOPTM phosphatase inhibitor (Roche). Protein separation was performed by SDS-page using Bolt 4 to 12% Bis-Tris protein gels (Invitrogen). Transferences were done in an iBlot 2 gel transfer device using iBlot 2 transfer nitrocellulose stacks (Invitrogen). Membranes were blotted against phospho-AKT (Ser473) (Cell Signaling), phospho-RAPTOR (Ser792) (Cell Signaling), phospho-p70 S6 Kinase (Thr389) (Cell Signaling), β-ACTIN (Cell Signaling) and their correspondent HRP-conjugated secondary antibodies. The revelation was performed using the SuperSignal West Femto Maximum Sensitivity Substrate (Thermo Scientific) and images were acquired using an ImageQuant LAS 4000 (GE Healthcare).

### Flow cytometry

Single-cell suspensions were stained and analyzed using a FACS LSR Fortessa™ system (BD Biosciences). Dead cells were excluded with the LIVE/DEAD Fixable Violet Dead Cell staining kit (Life technologies). The cells were then incubated for 10 minutes with purified rat anti-mouse CD16/CD32 (Biolegend, 93 clone)(only for mouse cells) and normal mouse serum (Interchim) before being stained with various monoclonal antibodies for 20 minutes in the dark on ice. For mouse cells, lineage-positive cells were excluded using the PerCP5.5-conjugated anti-CD3 (17A2), anti-NK1.1 (PK136), anti-CD19 (6D5), anti-Ly6G (1A8) (Biolegend). PerCP-conjugated anti-CCR3 (83103) added to the lineage staining to exclude eosinophils was from R&D. Alexa Fluor 700-conjugated anti-Ly6C (AL21) was from BD Pharmingen. PECF594-conjugated anti-CD11c (HL3) was from BD Horizon. Allophycocyanin-Cy7-conjugated anti-CD11b (M1/70), Brilliant violet 510-conjugated anti-MHC Class II (I-A/I-E) (M5/114.15.2), Brilliant violet 650-conjugated anti-CD45.2 (104), Brilliant violet 711-conjugated anti-CD45.1 (A20), PE-conjugated anti-CD135 (A2F10), APC-conjugated anti-CD115 (AFS98), PE-conjugated anti-CD226 (10E5), FITC-conjugated anti-CD102 (3C4) were all from Biolegend. PE-conjugated anti-CCR2 (475301) was from R&D systems. The data were analyzed with Flowjo software V10.1 (TreeStar). For human cells, a similar procedure was used with anti-HLA-DR FITC (eBioscience, clone LN3), anti-CD1a APC (Biolegend, clone HI149), anti-CD16 PE-Cy7 (BD Pharmingen, clone 3G8), anti-CD14 PE (Miltenyi Biotec, clone REA599).

### THP1 cell culture and stimulation

The THP1 monocytic cell line was cultured in RPMI 1640 medium (Gibco) supplemented with 10% heat-inactivated FBS (Gibco), L-glutamine (Thermo Fisher), Penicillin and Streptomycin (Thermo Fisher), MEM non-essential amino acids (Thermo Fisher), sodium pyruvate (Thermo Fisher), HEPES (Thermo Fisher) and 0.05mM of 2-mercaptoethanol (Thermo Fisher). Cells were kept in the culture at cell concentrations ranging from 2x10^5^ cells/mL to 8x10^5^ cells/mL and routinely verified negatively for mycoplasma contamination by PCR analysis. THP1 stimulation was performed in 96-well flat bottom plates at 1x10^5^ cells per well in a final volume of 200 μl. Cells were stimulated with two sequential treatments of 24 hours each. For the first 24 hours of treatment, cells were cultured in RPMI complete medium or RPMI medium with MDP at 100μg/ml. The second 24-hour treatment consisted of LPS (Invivogen) at 50ng/ml or RPMI medium. In selected experiments, the first treatment was MHY1485 (Sigma) at 2μM, rapamycin (Sigma) at 100nM, or wortmannin (Sigma) at 1μM. In some conditions, THP-1 macrophages were generated by adding PMA (5ng/ml) for 48h, followed by at least 2 days without PMA (121, 122). Culture supernatants were collected after the second treatment and TNF-α levels were quantified by ELISA using the Human TNF-alpha DuoSet ELISA (R&D systems) following manufacturer recommendations.

### Generation of BM-derived dendritic cells

BM cells were flushed out of the mouse bones with complete RPMI 1640 (Gibco). A single-cell suspension was then prepared by repeated pipetting. BM-derived dendritic cells (BMDCs) were generated for 7 days in respectively RPMI-1640 medium (Gibco), supplemented with glutamine, penicillin, streptomycin, 2-mercaptoethanol ([all from Gibco)], and 10% heat-inactivated fetal calf serum (GE Healthcare). The medium was supplemented with 20 ng/ml GM-CSF of J558 cells (GM-CSF-producing cells). Half of the medium was removed on day 3 and a new medium supplemented with GM-CSF supernatant (2x, 40 ng/ml) was added (55).

### Extracellular acidification rate (ECAR)

ECAR was measured under basal conditions and in response to glucose (10mM) using the Seahorse Glycolysis Stress Test Kit by using a Seahorse bioanalyser.

### Gene expression

RNAs were extracted using the RNEasy mini kit (Qiagen). According to the manufacturer’s instructions, isolated RNA was reverse-transcribed with the cDNA synthesis kit (Agilent Technologies). The resulting cDNA (equivalent to 500ng of total RNA) was amplified using the SYBR Green real-time PCR kit and detected on a Stratagene Mx3005 P (Agilent Technologies). qPCR was conducted using forward and reverse primers (sequences available upon request). The relative abundance of gene expression was assessed using the 2−ΔΔCt method. Actb was used as an internal reference gene in order to normalize the transcript levels.

### Statistics

Data were analyzed using Prism6.0 (GraphPad Software, San Diego, CA). Statistical significance was assessed by non-parametric Mann-Whitney test or two-way ANOVA for multiple comparisons. Values represent the mean of normalized data ± SEM. *, P<0.05; **, P<0.01; ***, P<0.001; ****, P<0.0001.

## Data availability statement

Publicly available datasets were analyzed in this study. This data can be found here: https://www.ncbi.nlm.nih.gov/geo/query/acc.cgi?acc=GSE69446: GEO database (accession number GSE69446).

## Ethics statement

All animal studies were approved by the local investigational review board of the Institut Pasteur of Lille (N°28010-2016012820187595).

## Author contributions

Conceptualization: CC, DAS, ES, MC, and LP. Methodology: CC, DAS, KR, OB, WL, MD, NW, ES, JK, MC, and LP. Formal analysis: CC, DAS, KR, OB, WL, NW, ES, JK, MC, and LP. Investigation: CC, DAS, KR, OB, WL, MD, NW, JK, MC, and LP. Writing – original draft: MC and LP. Writing – review and editing: all authors. Visualization: CC, DAS, MC, and LP. Supervision: LP and MC. Funding acquisition: MC and LP. All authors contributed to the article and approved the submitted version.

## Supplementary material

Supplementary Figure 1 Mixed BM chimera mice were generated as described in . Absolute numbers **(A)** and frequency **(B)** and their relative ratios of total WT and Nod2^-/-^ mo-DCs and mo-Macs in the colon of recipients are depicted and the ratio of mo-DCs vs mo-Macs (n=4). Bars indicate mean ± SEM. Statistical significance was assessed by non-parametric Mann-Whitney test. * P<0.05.

Supplementary Figure 2 Gating strategy of the mixed BM chimera mice generated as described in . **(A)** Contour plots of conventional DC1, DC2, mo-DCs, CD11c^+^ Macs, mo-Macs, Monocyte MHCII^+^ and Monocyte MHCII^-^ are depicted. **(B)** Each subset is represented according to CD45.1 and CD45.2. **(C)** MHCII GeoMean in Monocyte MHCII^+^.

Supplementary Figure 3 Gating strategy in WT GF **(A)**, in *Nod2* ^-/-^ SPF **(B)**, and in *Nod2* ^-/-^ GF (C) as explained in . Contour plots and frequency of conventional DC1, DC2, of mo-DCs, mo-Macs, and Monocyte MHCII+ are depicted.

Supplementary Figure 4 Fecal microbiota from WT mice was transplanted in GF mice that are deficient or not for *Nod2*. Four weeks after colonization, the proportions of mononuclear phagocytes were evaluated in the transplanted mice. **(A)** Experimental set-up. **(B)** Frequency and absolute number of CD11c^-^ MHCII^+^ and CD11c^+^ MHCII+ cells (n=3/group).

Supplementary Figure 5 Expression of *NOD2* in mo-DCs upon differentiation for 5 days with GM-CSF and IL-4. Bars indicate mean ± SEM from three biological replicates. The statistical significance was assessed by multi-comparison non-parametric Friedman paired test, with Dunn’s post-test. *, P<0.05, **, P<0.01.

Supplementary Figure 6
**(A)** THP1 (left part) and THP1 *NOD2*-deficient cells (right part) were stimulated for the indicated time with MDP and the presence of phosphorylated RAPTOR (Ser792) was measured at 0, 30min, and 60min by western blot. β-ACTIN was measured as a control. **(B)** THP1 (left part) and THP1 *NOD2*-deficient cells (right part) were stimulated for the indicated time with MDP, LPS, or both sequentially for 24 hours each and the presence of phosphorylated AKT (Ser473), and phospho-p70 S6 Kinase (Thr389) was measured at different timepoints by western blot. β-ACTIN was measured as a control. Quantification of the western-blots **(C)** *IRF4* and *IRF8* mRNA expression in THP1 (blue) and THP1 NOD2^-/-^ (red) monocytic cell lines was measured by RT-qPCR at the beginning of the culture or 24h and 48h after MDP treatment. Data are representative of 2 independent experiments with at least three biological replicates. Bars indicate mean ± SEM. Statistical significance was assessed by ordinary one-way multiple comparisons **(A)**. ***, P<0.005.

Supplementary Figure 7 MDP enhances the differentiation of Mo-DCs in a glycolytic and MTORC1-independent manner. **(A)** Overexpressed enzymes involved in glycolysis in MDP-treated mouse monocytes in published RNA-seq data sets (GEO accession number GSE101496). **(B)** Mitochondrial respiration calculated as OCR (B, upper part), glycolysis activity calculated as ECAR (B, lower part), before and after Glc (glucose) administration. (Oligo, oligomycin; 2DG, 2-deoxyglucose; AA, antimycin A; Rot, rotenone). Extracellular acidification rate (ECAR) was measured in the MDP-treated *Nod2* ^+/+^ (blue) and *Nod2* ^-/-^ THP1 (red) cells. Data are representative of 2 independent experiments with at least four biological replicates. **(C)** Measure of the ECAR non glycolytic, ECAR basal and ECAR maximal between the THP1 *NOD2* ^+/+^ and *NOD2* ^-/-^.

Supplementary Figure 8 Mouse BM monocytes from WT **(A)** and *Nod2* ^-/-^ mice **(B)** were treated with MDP or LPS for 24h (1^st^ Tx), and washed before a second treatment with MDP or LPS (2^nd^ Tx). mTNF-α was measured 24h after the last treatment in the supernatant. Data are representative of 2 independent experiments with at least three biological replicates. Bars indicate mean ± SEM. Statistical significance was assessed by the non-parametric Mann-Whitney test. **, P<0.01.

Supplementary Figure 9 Mo-DCs were differentiated in the presence of MDP, and hTNF-α was measured at 24h. Bars indicate the mean ± SEM of four biological replicates. Statistical significance was assessed by the non-parametric Mann-Whitney test. *, P<0.05.
