# Supplementary figures and images for: NOD2 in monocytes negatively regulates macrophage development through TNFalpha
Source: Front Immunol. 2023 Jun 21;14:1181823. doi: 10.3389/fimmu.2023.1181823 (PMC10320732; doi:10.3389/fimmu.2023.1181823)

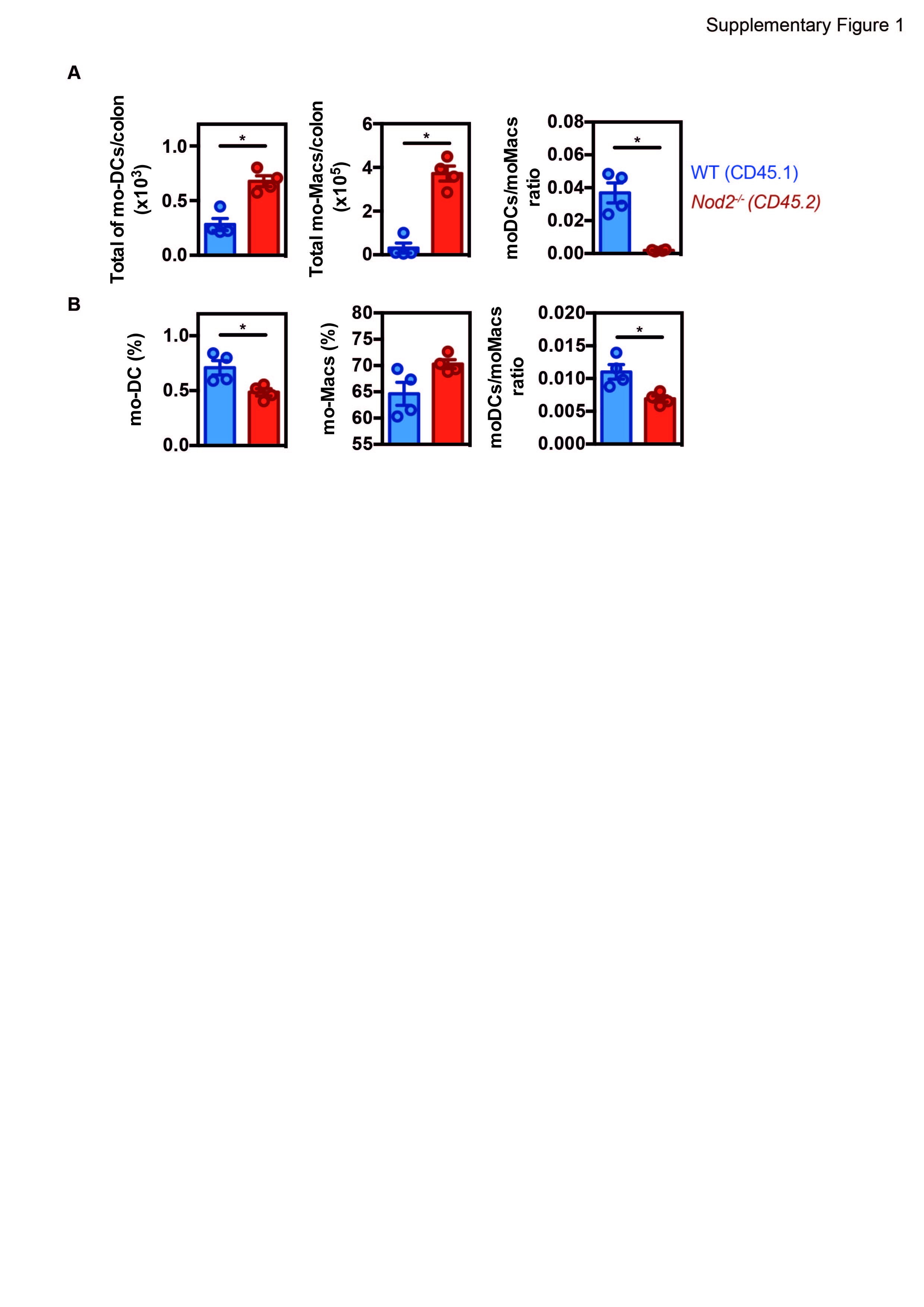

Supplement: Supplementary Figure 1 — Mixed BM chimera mice were generated as described in . Absolute numbers (A) and frequency (B) and their relative ratios of total WT and Nod2-/- mo-DCs and mo-Macs in the colon of recipients are depicted and the ratio of mo-DCs vs mo-Macs (n=4). Bars indicate mean ± SEM. Statistical significance was assessed by non-parametric Mann-Whitney test. * P<0.05. [file Image_1.jpeg]

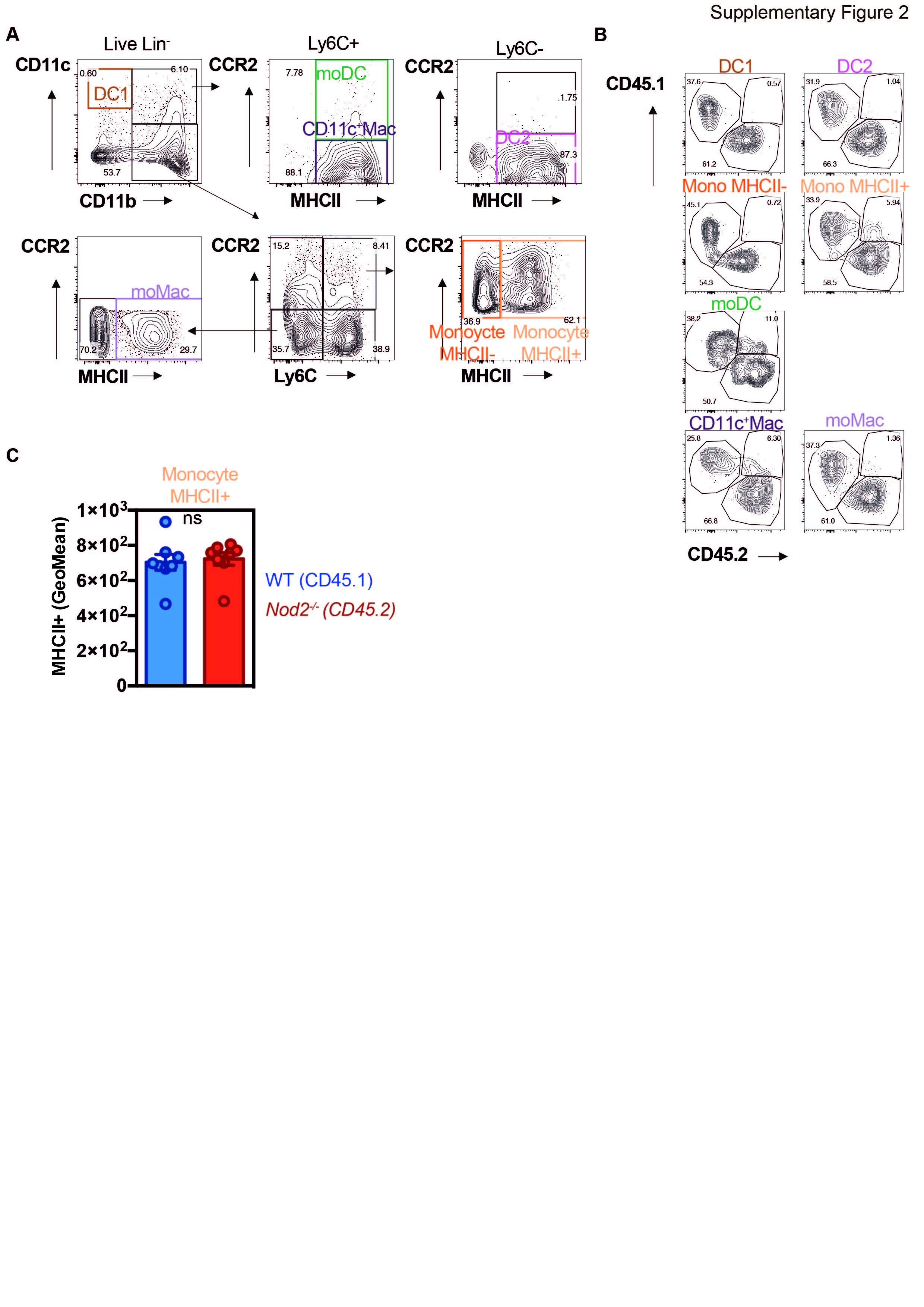

Supplement: Supplementary Figure 2 — Gating strategy of the mixed BM chimera mice generated as described in . (A) Contour plots of conventional DC1, DC2, mo-DCs, CD11c+ Macs, mo-Macs, Monocyte MHCII+ and Monocyte MHCII- are depicted. (B) Each subset is represented according to CD45.1 and CD45.2. (C) MHCII GeoMean in Monocyte MHCII+. [file Image_2.jpeg]

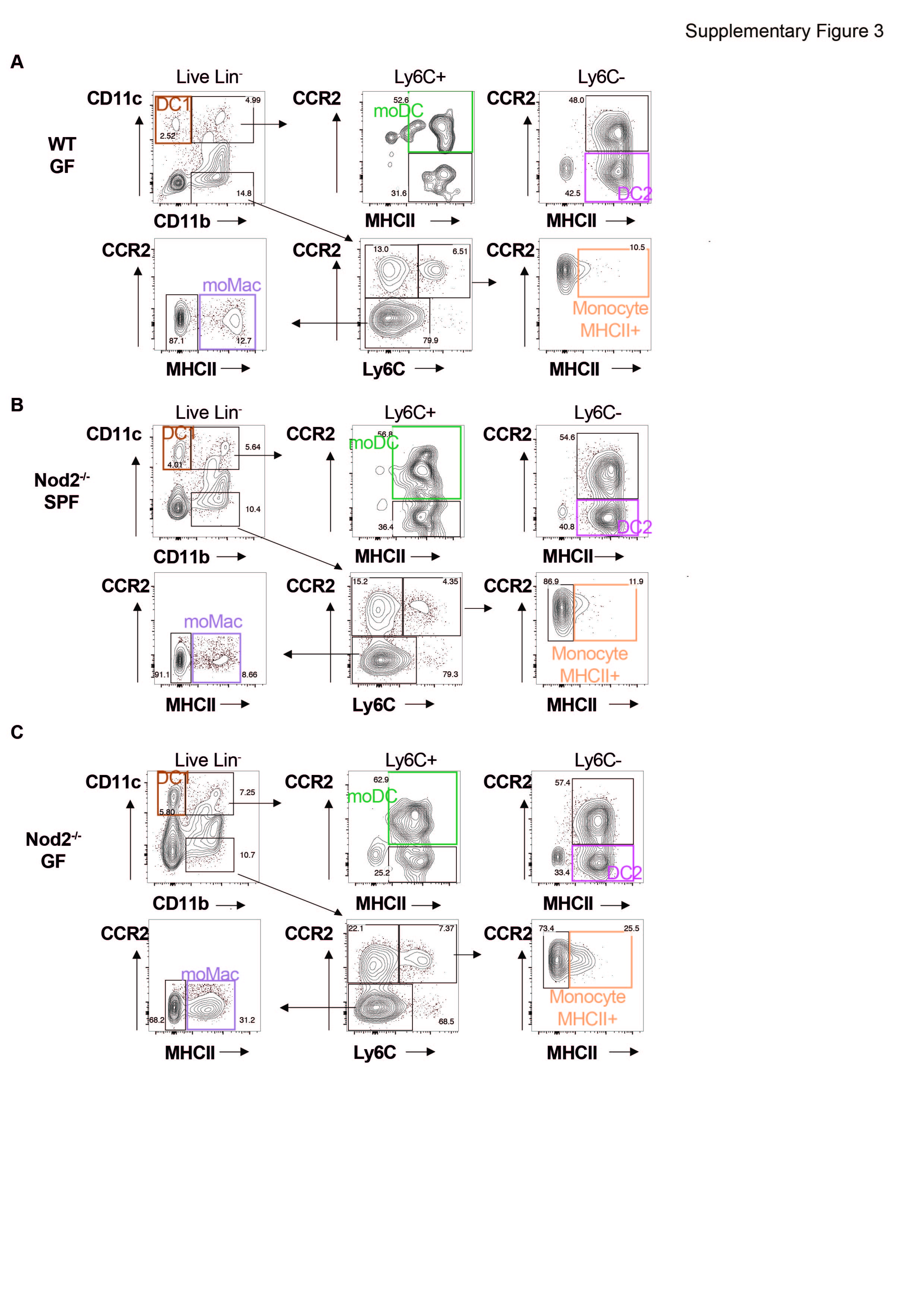

Supplement: Supplementary Figure 3 — Gating strategy in WT GF (A), in Nod2 -/- SPF (B), and in Nod2 -/- GF (C) as explained in . Contour plots and frequency of conventional DC1, DC2, of mo-DCs, mo-Macs, and Monocyte MHCII+ are depicted. [file Image_3.jpeg]

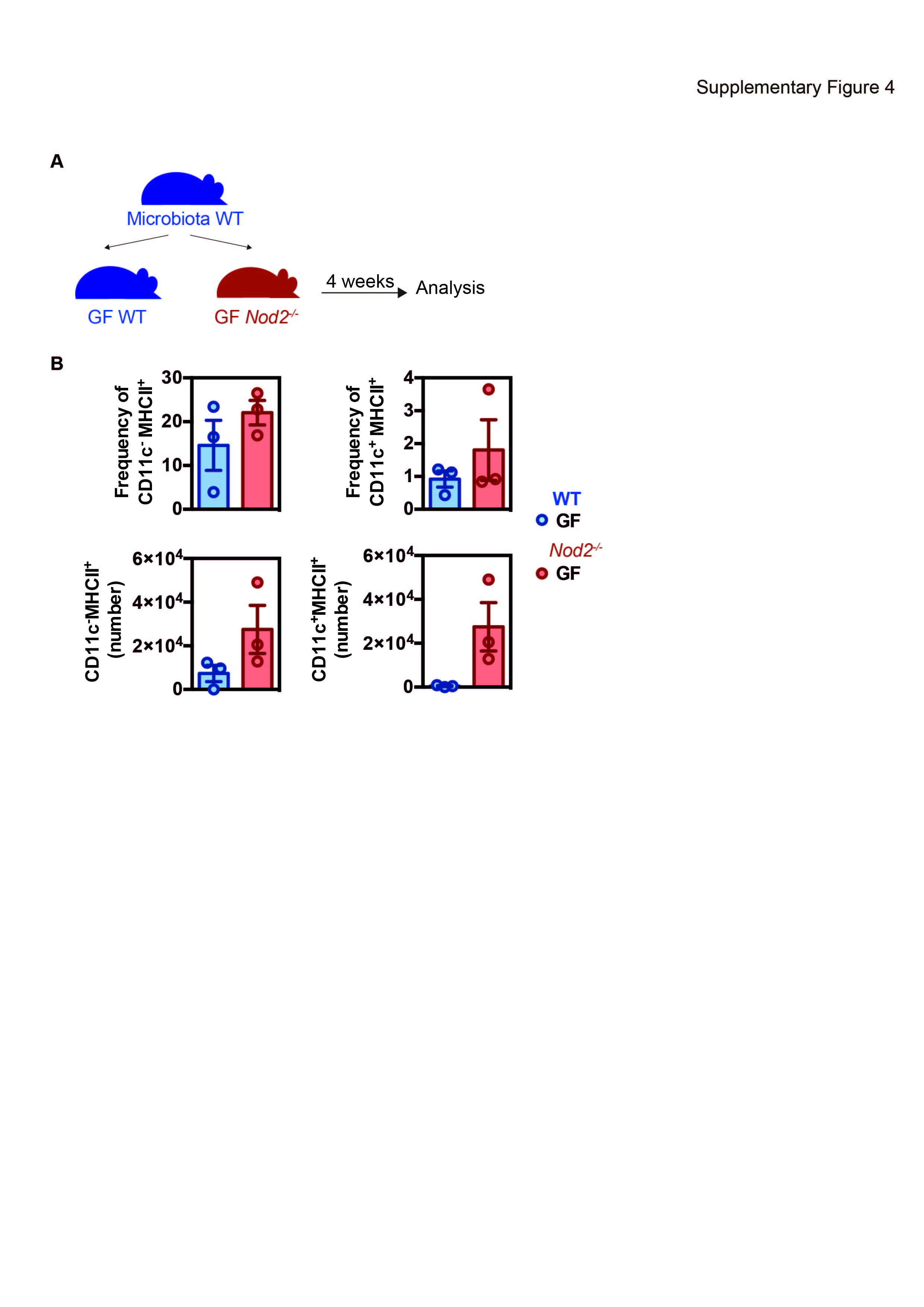

Supplement: Supplementary Figure 4 — Fecal microbiota from WT mice was transplanted in GF mice that are deficient or not for Nod2. Four weeks after colonization, the proportions of mononuclear phagocytes were evaluated in the transplanted mice. (A) Experimental set-up. (B) Frequency and absolute number of CD11c- MHCII+ and CD11c+ MHCII+ cells (n=3/group). [file Image_4.jpeg]

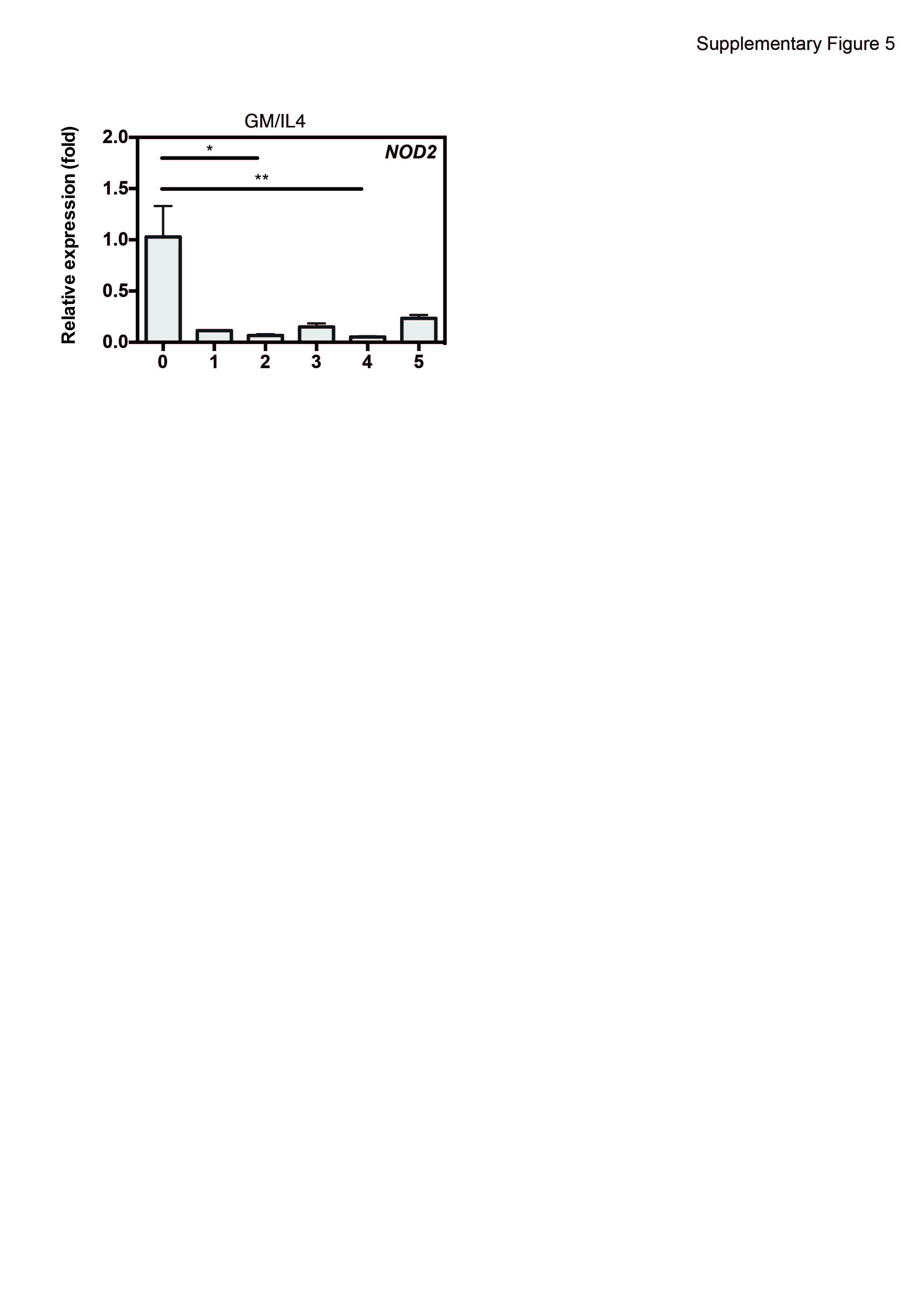

Supplement: Supplementary Figure 5 — Expression of NOD2 in mo-DCs upon differentiation for 5 days with GM-CSF and IL-4. Bars indicate mean ± SEM from three biological replicates. The statistical significance was assessed by multi-comparison non-parametric Friedman paired test, with Dunn’s post-test. *, P<0.05, **, P<0.01. [file Image_5.jpeg]

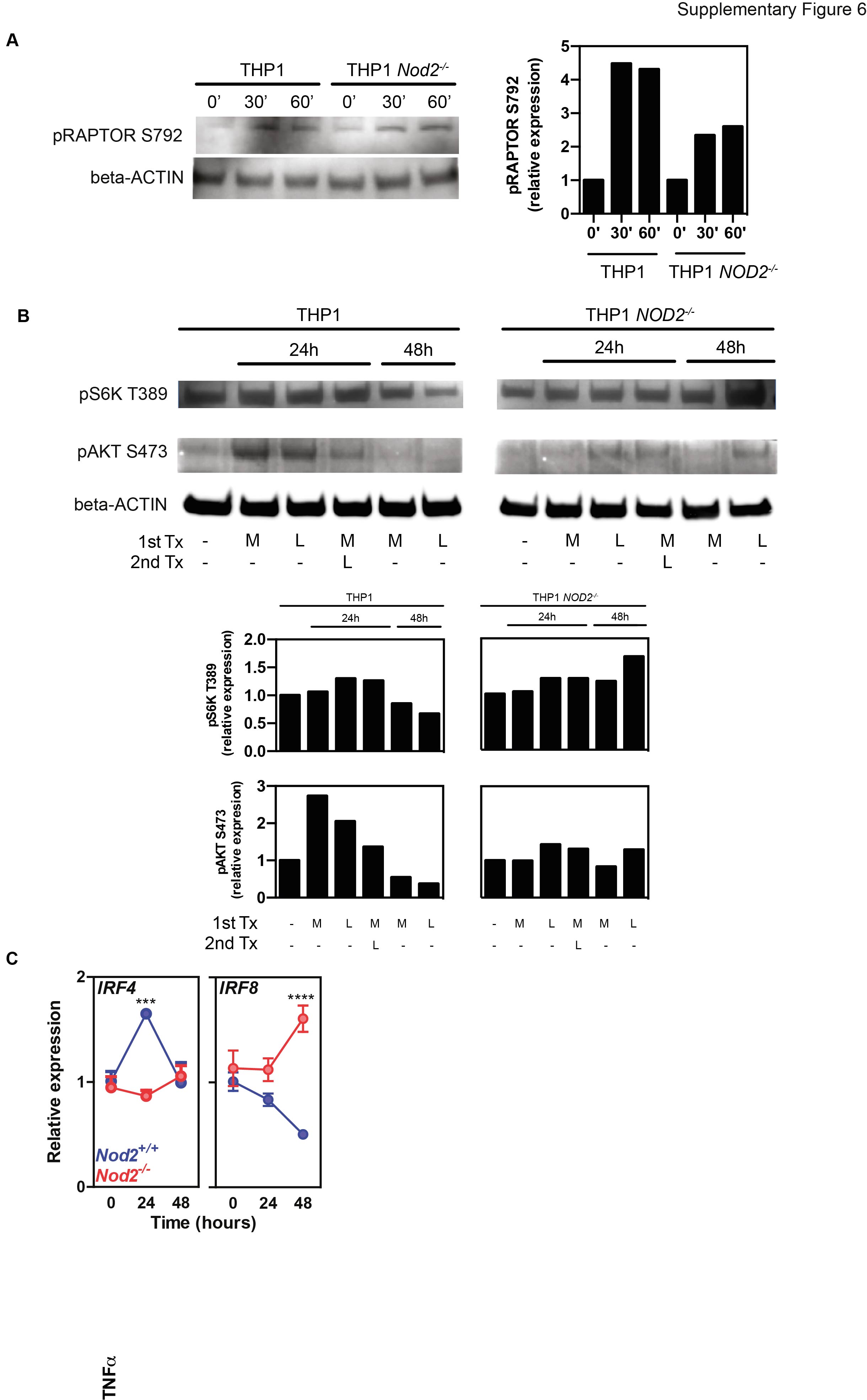

Supplement: Supplementary Figure 6 — (A) THP1 (left part) and THP1 NOD2-deficient cells (right part) were stimulated for the indicated time with MDP and the presence of phosphorylated RAPTOR (Ser792) was measured at 0, 30min, and 60min by western blot. β-ACTIN was measured as a control. (B) THP1 (left part) and THP1 NOD2-deficient cells (right part) were stimulated for the indicated time with MDP, LPS, or both sequentially for 24 hours each and the presence of phosphorylated AKT (Ser473), and phospho-p70 S6 Kinase (Thr389) was measured at different timepoints by western blot. β-ACTIN was measured as a control. Quantification of the western-blots (C) IRF4 and IRF8 mRNA expression in THP1 (blue) and THP1 NOD2-/- (red) monocytic cell lines was measured by RT-qPCR at the beginning of the culture or 24h and 48h after MDP treatment. Data are representative of 2 independent experiments with at least three biological replicates. Bars indicate mean ± SEM. Statistical significance was assessed by ordinary one-way multiple comparisons (A). ***, P<0.005. [file Image_6.jpeg]

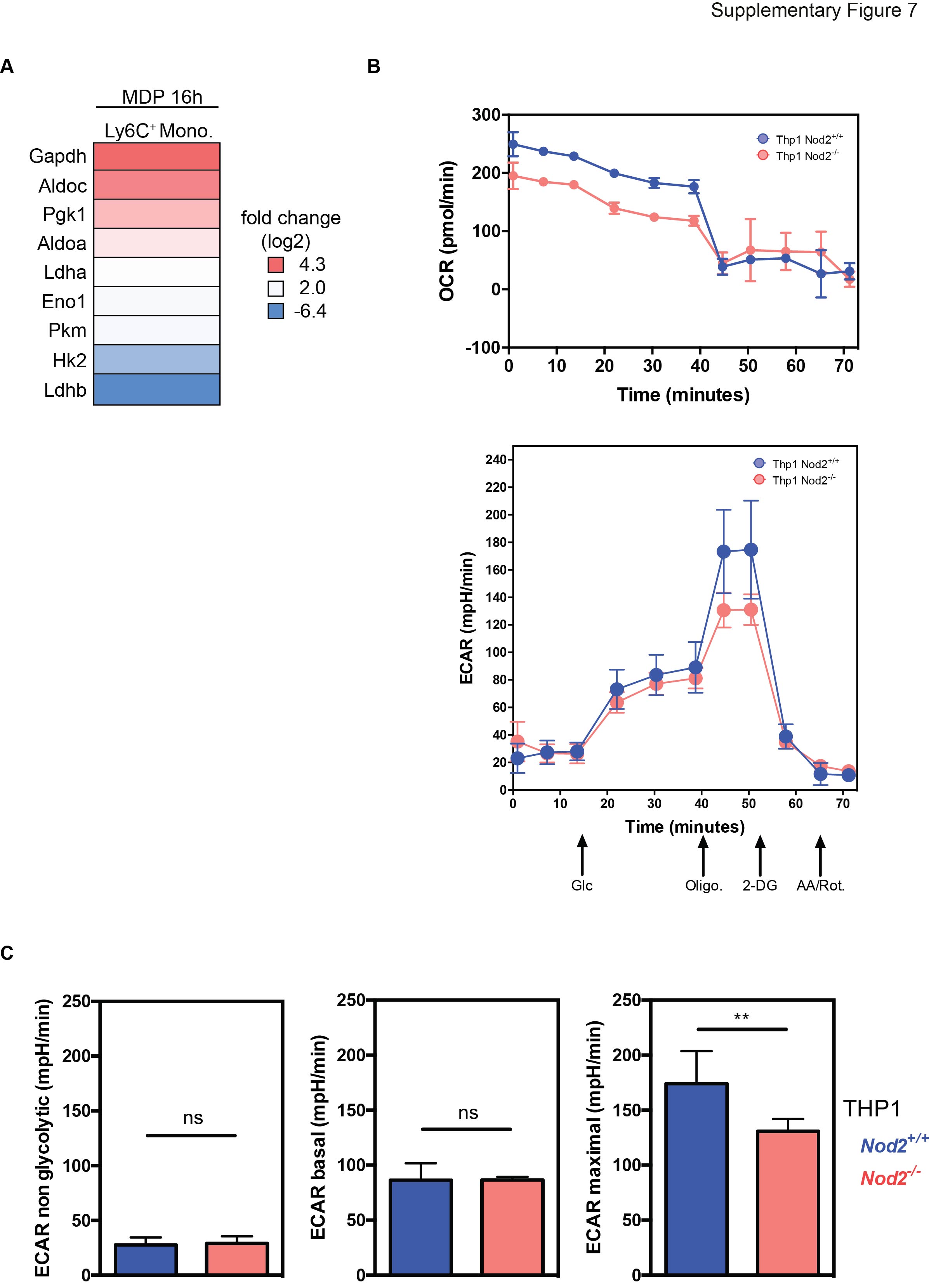

Supplement: Supplementary Figure 7 — MDP enhances the differentiation of Mo-DCs in a glycolytic and MTORC1-independent manner. (A) Overexpressed enzymes involved in glycolysis in MDP-treated mouse monocytes in published RNA-seq data sets (GEO accession number GSE101496). (B) Mitochondrial respiration calculated as OCR (B, upper part), glycolysis activity calculated as ECAR (B, lower part), before and after Glc (glucose) administration. (Oligo, oligomycin; 2DG, 2-deoxyglucose; AA, antimycin A; Rot, rotenone). Extracellular acidification rate (ECAR) was measured in the MDP-treated Nod2 +/+ (blue) and Nod2 -/- THP1 (red) cells. Data are representative of 2 independent experiments with at least four biological replicates. (C) Measure of the ECAR non glycolytic, ECAR basal and ECAR maximal between the THP1 NOD2 +/+ and NOD2 -/-. [file Image_7.jpeg]

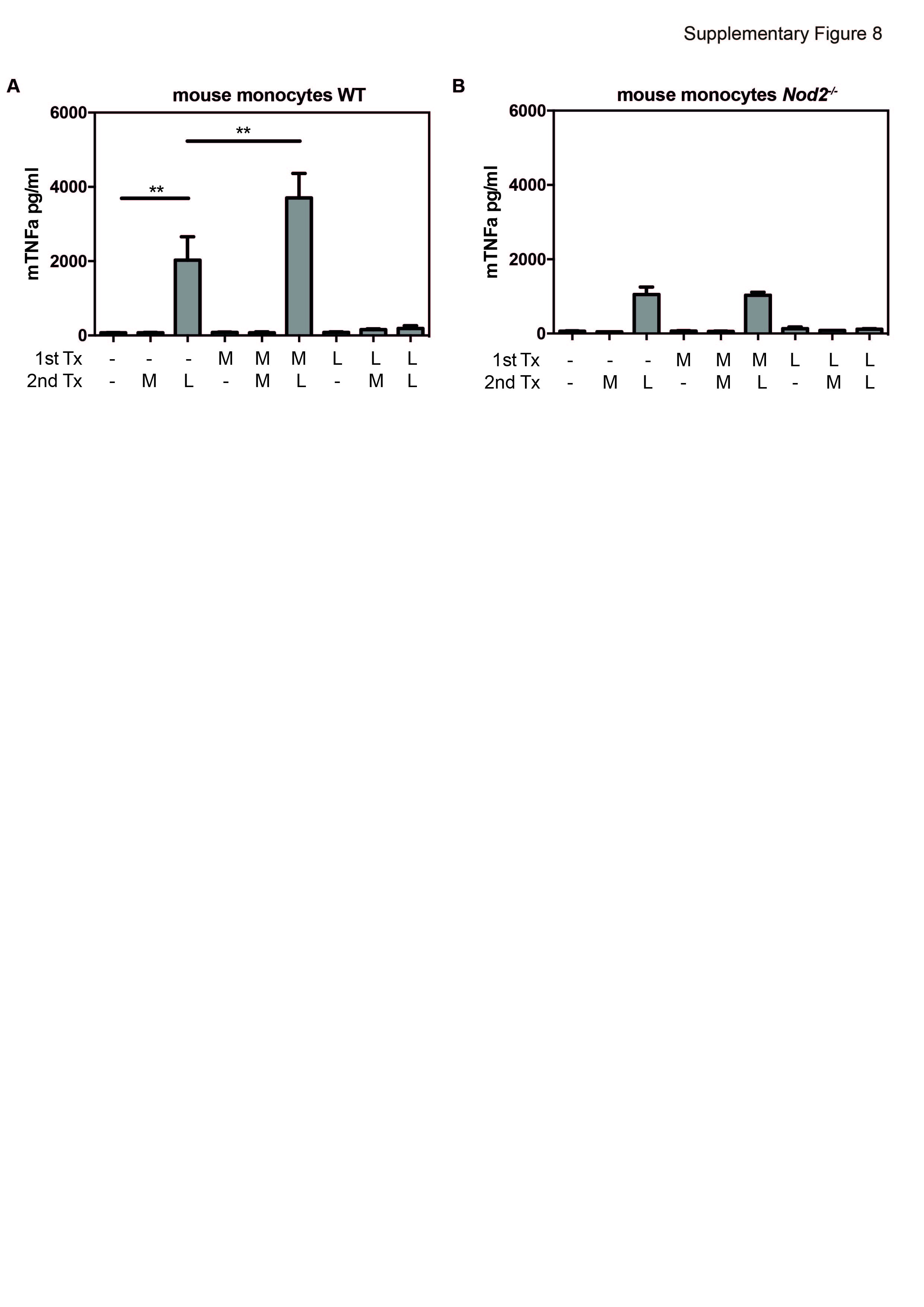

Supplement: Supplementary Figure 8 — Mouse BM monocytes from WT (A) and Nod2 -/- mice (B) were treated with MDP or LPS for 24h (1st Tx), and washed before a second treatment with MDP or LPS (2nd Tx). mTNF-α was measured 24h after the last treatment in the supernatant. Data are representative of 2 independent experiments with at least three biological replicates. Bars indicate mean ± SEM. Statistical significance was assessed by the non-parametric Mann-Whitney test. **, P<0.01. [file Image_8.jpeg]

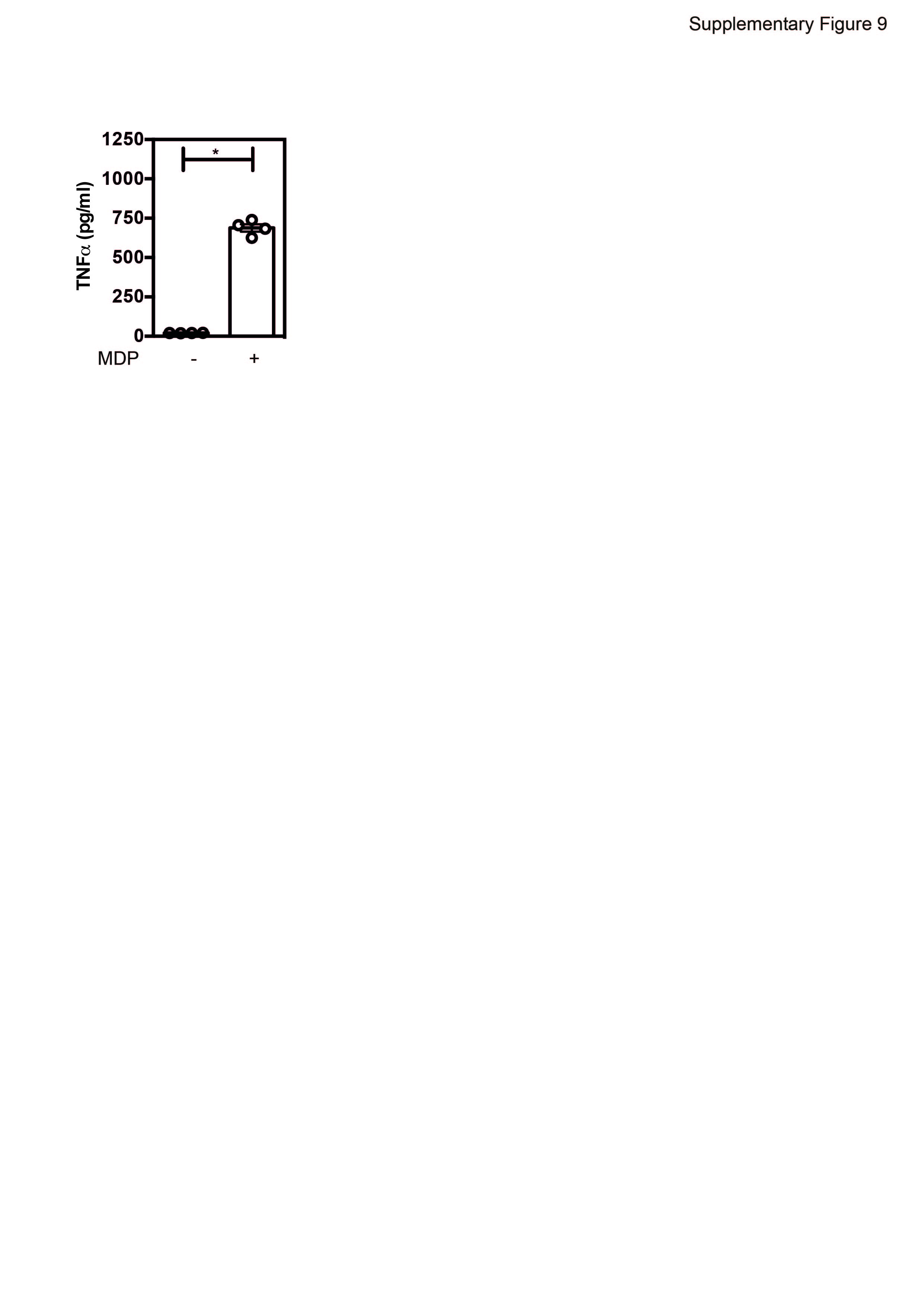

Supplement: Supplementary Figure 9 — Mo-DCs were differentiated in the presence of MDP, and hTNF-α was measured at 24h. Bars indicate the mean ± SEM of four biological replicates. Statistical significance was assessed by the non-parametric Mann-Whitney test. *, P<0.05. [file Image_9.jpeg]
